# The impact of violating the independence assumption in meta-analysis on biomarker discovery

**DOI:** 10.3389/fgene.2022.1027345

**Published:** 2023-01-04

**Authors:** Farnoosh Abbas-Aghababazadeh, Wei Xu, Benjamin Haibe-Kains

**Affiliations:** ^1^ Princess Margaret Cancer Centre, University Health Network, Toronto, ON, Canada; ^2^ Department of Medical Biophysics, University of Toronto, Toronto, ON, Canada; ^3^ Dalla Lana School of Public Health, University of Toronto, Toronto, ON, Canada; ^4^ Ontario Institute for Cancer Research, Toronto, ON, Canada; ^5^ Department of Computer Science, University of Toronto, Toronto, ON, Canada

**Keywords:** meta-analysis, non-independent effects, gene expression, pharmacogenomics, biomarker

## Abstract

With rapid advancements in high-throughput sequencing technologies, massive amounts of “-omics” data are now available in almost every biomedical field. Due to variance in biological models and analytic methods, findings from clinical and biological studies are often not generalizable when tested in independent cohorts. Meta-analysis, a set of statistical tools to integrate independent studies addressing similar research questions, has been proposed to improve the accuracy and robustness of new biological insights. However, it is common practice among biomarker discovery studies using preclinical pharmacogenomic data to borrow molecular profiles of cancer cell lines from one study to another, creating dependence across studies. The impact of violating the independence assumption in meta-analyses is largely unknown. In this study, we review and compare different meta-analyses to estimate variations across studies along with biomarker discoveries using preclinical pharmacogenomics data. We further evaluate the performance of conventional meta-analysis where the dependence of the effects was ignored *via* simulation studies. Results show that, as the number of non-independent effects increased, relative mean squared error and lower coverage probability increased. Additionally, we also assess potential bias in the estimation of effects for established meta-analysis approaches when data are duplicated and the assumption of independence is violated. Using pharmacogenomics biomarker discovery, we find that treating dependent studies as independent can substantially increase the bias of meta-analyses. Importantly, we show that violating the independence assumption decreases the generalizability of the biomarker discovery process and increases false positive results, a key challenge in precision oncology.

## 1 Introduction

Patient response to anticancer drugs varies widely, and the genomic-make-up of a patient’s tumor is a major factor contributing to this variation. The filed of pharmacogenomics studies the influence of genomic variation on individualized drug response (Iorio et al., 2016). Due to the limited access and high cost of clinical samples, cancer cell lines are frequently used to investigate disease biology and its relationship to drug response for biomarker discovery (Basu et al., 2013; Iorio et al., 2016).

Due to the complexity of generating pharmacogenomic datasets, the reproducibility of preclinical data and the findings from high-throughput profiling studies in cancer cell lines has been extensively investigated (Haibe-Kains et al., 2013; Haverty et al., 2016; Safikhani et al., 2016). Technological improvements in high-throughput drug screening enable the generation of large-scale pharmacogenomic datasets, which provide a remarkable opportunity for the identification of biomarkers predictive of drug response. However, biomarkers often fail to generalize across independent studies. This is due to the complexity of biological systems, the use of different experimental protocols, and the application of various technology platforms for both molecular profiling and data processing methods (Haibe-Kains et al., 2013; Haverty et al., 2016; Dempster et al., 2019). Moreover, the large number of features and relatively small number of cell lines can lead to findings that are not generalizable and show bias when assessed in independent cohorts (Tseng et al., 2012; Hatzis et al., 2014; Sweeney et al., 2017).

To address these issues, meta-analyses can be performed to integrate independent studies to identify more reliable biomarkers by increasing statistical power and reducing false positives (Sweeney et al., 2017; Abbas-Aghababazadeh et al., 2020; Borenstein et al., 2021). In recent years, several meta-analysis methods have been proposed such as combining *p*-values (Fisher et al., 1934; Stouffer et al., 1949; Won et al., 2009), combining effect estimates (Borenstein et al., 2021), and rankings (Hong et al., 2006). The strengths and limitations of meta-analyses are evaluated particularly with respect to their ability to assess variation across studies or heterogeneity (e.g., platform variability, inconsistent annotation, various methods for data processing, cell lines heterogeneity, and laboratory-specific effects) beyond within-study variation (e.g., experimental designs and populations of interest) (Veroniki et al., 2016). As a result, the selection of an optimal meta-analysis method depends considerably on the available data structure and the hypothesis setting to achieve the underlying biological goal (Chang et al., 2013).

Combining the *p*-values from multiple independent studies has the benefit of simplicity and extensibility to different kinds of outcome variables (Tseng et al., 2012; Chang et al., 2013). However, this can only be performed under the parametric assumption where the *p*-values are uniformly distributed under the null hypothesis, while not accounting for the data heterogeneity and direction of effect sizes, which represents a major limitation of this method (Marot et al., 2009). Approaches that combine effects including fixed- and random-effects (FE & RE) models are widely used to achieve a broad inferential basis for evaluations of effects (Cochran, 1954; Borenstein et al., 2021). Under the FE model, we assume that there is one true effect that underlies all the studies in the analysis, and that all differences in observed effects are due to sampling error. In contrast, the RE model incorporates the variability of the effects across studies in addition to the within-study variability using a two-stage hierarchical process. Several approaches have been proposed to combine individual study results into an overall estimate of effect using the inverse-variance strategy (Egger et al., 2008).

Assessing heterogeneity is a critical issue in meta-analysis because different models may lead to different estimates of overall effect and different standard errors. Several approaches have been suggested that vary in popularity and complexity for how best to carry out such combinations (Veroniki et al., 2016; Guolo and Varin, 2017; Langan et al., 2019). Cochran (Cochran, 1954) proposed a *Q* test to determine the heterogeneity across studies, however its statistical power depends on the number of studies and sample size (Whitehead and Whitehead, 1991; Turner et al., 2012; Hoaglin, 2016; Bodnar et al., 2017). Higgins and Thompson proposed better statistic *I* ^2^ to describe heterogeneity that reflects the proportion of total variance that is attributed to heterogeneity (Higgins et al., 2003). Meta-analysis allows us to quantify heterogeneity between studies, but precisely estimating the between-study heterogeneity is challenging. This is especially true if the number of studies included is small. A Bayesian approach was proposed to capture the uncertainty in the estimation of the between-study variance by incorporating prior knowledge (Sutton and Abrams, 2001; Bodnar et al., 2017; Röver, 2017).

Traditional meta-analysis procedures make a crucial assumption: effects are independent. When this assumption is violated, conclusions based on meta-analyses can be misleading and will bias the overall effect estimate along with inflating Type I error. In real-world applications, there are various sources of effect dependencies within and across studies. For instance, a meta-analysis uses more than one outcome measure for the same sample of participants, two treatment groups with the same control group, duplicate full or partial data or samples, and effects reported by the same research group (Becker, 2000; Wood, 2008; Lin and Sullivan, 2009; Hedges et al., 2010; Cheung, 2014; Scammacca et al., 2014; Van den Noortgate et al., 2015; Cheung, 2019; Cooper et al., 2019; Liu et al., 2019; Wilson, 2019; Liu and Xie, 2020; Luo et al., 2020; Borenstein et al., 2021). The dependence of the effects must be resolved in a way that permits each study to contribute a single independent effect to the meta-analysis or modeled dependence in order to avoid threats to the validity of the meta-analysis results. Reflecting on duplicate data problems, detecting potential duplicate studies and options for the correction of duplication were discussed, while the issue of prevention of duplication has not yet been addressed (Wood, 2008). In general, different strategies have been proposed to handle dependency including ignoring dependence, avoiding dependence [e.g., averaging effects (Wood, 2008) or shifting unit-of-analysis (Cooper et al., 2019)], modeling dependence [e.g., robust variance estimation (Hedges et al., 2010), multivariate meta-analysis (Becker, 2000), multilevel meta-analysis (Cheung, 2014; Van den Noortgate et al., 2015; Cheung, 2019)], and determining the covariance of effects across studies for overlap samples (Lin and Sullivan, 2009; Luo et al., 2020). Moreover, the Cauchy combination test Liu et al. (2019); Liu and Xie (2020) and the harmonic mean *p*-value Wilson (2019) were proposed as robust choices to combine *p*-values under arbitrary dependency structures to control type I error rate and improve power of analysis.

Several preclinical pharmacogenomic meta-analyses have been performed in an effort to discover predictive biomarkers with consistent evidence across multiple research laboratories (Cohen et al., 2011; Safikhani et al., 2016, 2017; Haverty et al., 2016; Ding et al., 2018; Jaiswal et al., 2021; Xia et al., 2022). However, virtually all existing datasets suffer from missing observations due to limitations of the experimental techniques, insufficient resolution, and sequencing costs which prevent complete profiling of cancer samples at the genomic and pharmacological levels (Choi and Pavelka, 2012; Basu et al., 2013; Muir et al., 2016; Li et al., 2017). Notably, the lack of either molecular (Basu et al., 2013) or pharmacological data [e.g., drug response Li et al. (2017)] in a given study prevents its use for biomarker discovery. Hence, investigators often attempt to make datasets more complete by borrowing a subset of the data (e.g., molecular data, drug response data, or combination of both) from one study and duplicating them in another study (Consortium of Drug Sensitivity in Cancer Consortium, 2015; Safikhani et al., 2016; Li et al., 2017; Ding et al., 2018; Jia et al., 2021; Xia et al., 2022). For instance, the re-analysis of drug response consistency by the Cancer Cell Line Encyclopedia and Genomics of Drug Sensitivity in Cancer, investigators duplicated the gene mutation, copy number alteration and mRNA expression when assessing the generalizability of biomarkers predictive of drug response (gene-drug associations) (Consortium of Drug Sensitivity in Cancer Consortium, 2015; Safikhani et al., 2016). In applied research, detection of non-independent effects or duplicate study effects along with modeling dependency remain challenging. When study effect estimates are non-independent, conclusions based on the conventional meta-analyses will bias the aggregated effects and can be misleading or even wrong (Wood, 2008; Cheung, 2019).

In this study, we reviewed and compared the performance of frequentist and Bayesian meta-analysis approaches to assess gene-drug associations or biomarker discovery using independent large-scale breast cancer and pan-cancer pharmacogenomic datasets. We found that changes in the number and size of studies along with the type of meta-analysis methods can affect the identification of statistically significant gene-drug associations. We further conducted simulation studies to assess the performance of including non-independent studies or effects in a traditional meta-analysis approach. Results showed that as the number of non-independent effects increased, higher relative mean squared error and lower coverage probability were observed. In addition, we aimed to evaluate the bias of avoiding the dependence of effects in traditional meta-analyses *via* preclinical pharmacogenomic data. To do so, we showed how increases in the number of duplicated studies can impact the bias of estimated overall effect and the identification of gene-drug associations. The results indicate that by increasing the number of dependent studies, bias of estimated overall effect may increase and genes with lower similarity of measured expression across studies denote higher bias.

## 2 Materials and Methods

### 2.1 Data types and sources

We used transcriptomic (RNA-Sequencing and gene expression microarray) and drug response data from pharmacogenomic cancer cell line sensitivity screenings, including the Cancer Cell Line Encyclopedia (CCLE: Broad-Novartis) (Barretina et al., 2012), the Genomics of Drug Sensitivity in Cancer (GDSC: Wellcome Trust Sanger Institute) (Garnett et al., 2012; Yang et al., 2012), the Genentech Cell Line Screening Initiative (gCSI) (Haverty et al., 2016), the Cancer Therapeutics Response Portal (CTRP: Broad Institute) (Basu et al., 2013), Oregon Health and Science University breast cancer screen (GRAY) (Heiser et al., 2012), and University Health Network Breast Cancer Screen (UHNBreast) (Marcotte et al., 2016; Safikhani et al., 2017) (Table 1).

**TABLE 1:** Studies used in meta-analyses and corresponding drug response versions, number of cell lines, and drugs along with molecular data type. Different versions of GDSC study refer to drug response assays along with updated cell lines and drugs. (*) represents the CCLE microarray data. Transcriptomic and drug response data were obtained from the *PharmacoGx* R package.

Dataset	Drug sensitivity	No. of cell lines	No. of drugs	Molecular data
**CCLE** Barretina et al. (2012)	2015	1,094	24	RNA-Seq
**CTRP** Basu et al. (2013)	2015	887	544	Microarray*
**GDSC1** Garnett et al. (2012)	2020 (v1-8.2)	1,060	343	Microarray
**GDSC2** Garnett et al. (2012)	2020 (v2-8.2)	1,104	190	Microarray
**gCSI** Haverty et al. (2016)	2017	747	16	RNA-Seq
**GRAY** Heiser et al. (2012)	2017	74	107	RNA-Seq
**UHNBreast** Safikhani et al. (2017)	2019	84	8	RNA-Seq

The cell lines’ gene expression profiles were generated using RNA sequencing (RNA-seq) in all datasets except for CTRP and GDSC datasets where the Affymetrix microarrays where used (Table 1). Molecular information was obtained from the *PharmacoGx* R package along with details on data processing (Safikhani et al., 2016; Smirnov et al., 2016). Cell line drug response data, in the form of area above the curve (AAC) recomputed information, was also obtained from the *PharmacoGx* R package (Table 1) (Smirnov et al., 2016).

**FIGURE 1:**
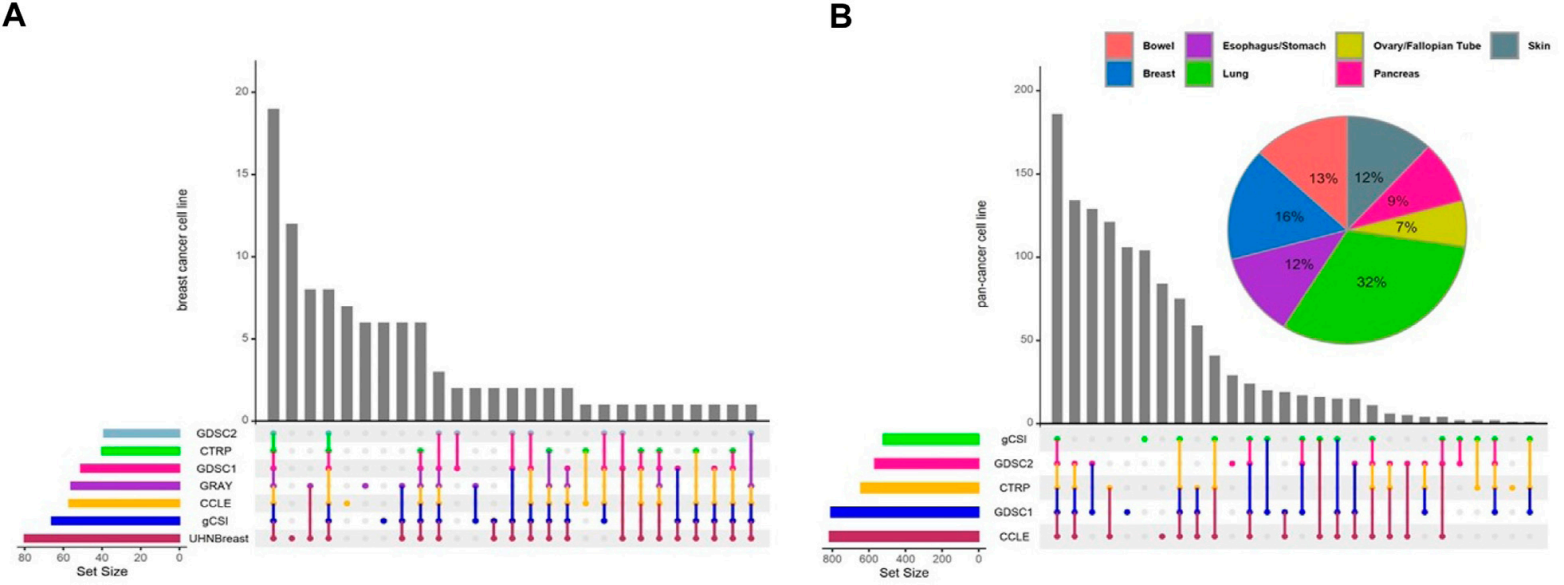
Distribution of cell lines across pharmacogenomic studies. Upset diagrams illustrate the number of cell lines for each study for **(A)** breast cancer and **(B)** pan-cancer data. Pie chart shows the distribution of common cell lines across tissue types using pan-cancer data. Tissue types contain at least 10 cell lines with available expression and drug response data included in pan-cancer analyses.

In this study, we performed a breast cancer-specific and a pan-cancer analysis to assess the generalization of our results. For the breast cancer analysis, we included seven independent studies, while the pan-cancer analysis excluded the UHNBreast and GRAY datasets as they included only breast cancer cell lines (Figure 1). Pan-cancer data was filtered for tissue types containing at least 10 cell lines with available expression and drug response data (Supplementary Figure S1). Breast cancer meta-analyses consisted of 11,198 genes and 19 cell lines shared between studies. To analyze pan-cancer data, we selected 11,340 common genes and 168 cell lines between studies. Common anticancer drugs such as Erlotinib, Lapatinib, and Paclitaxel across studies were used to evaluate gene-drug associations and discover biomarkers. Notably, Lapatinib and Erlotinib are a potent, oral, reversible, dual inhibitor of epidermal growth factor receptor (EGFR) and human epidermal growth factor receptor 2 (HER2 or ERBB2) for breast cancer and a few other solid tumors (Schlessinger, 2000; Geyer et al., 2006; Medina and Goodin, 2008; Iqbal et al., 2011). Additionally, Paclitaxel is a widely used drug for various solid tumors and breast cancer treatment, however, resistance occurs frequently and the evasion mechanisms remain unclear (Volk-Draper et al., 2014; Weaver, 2014).

### 2.2 Data pre-processing

The drug dose-response curves were summarized into AAC values using a 3-parameter logistic model as implemented in *PharmacoGx* (Smirnov et al., 2016). The AAC values range between 0 and 1, with AAC close to 1 indicating the drug sensitive cell lines and AAC close to 0 indicating the drug resistant cell lines. Missing drug response data (AAC values) were imputed within each study to simplify further analyses. Imputation was completed *via* multiple imputation by classification and regression trees (MI-CART) (Burgette and Reiter, 2010; Burgette and Reiter, 2010) as implemented in *mice* R package (Buuren and Groothuis-Oudshoorn, 2010).

For individual breast cancer and pan-cancer data analyses, we focused on genes and drugs that were measured in all studies. To avoid scaling issues and make comparisons between the estimated effects across studies, the *z*-score transformation (mean centered and variance scaled) was considered across cell lines using raw gene expression values and imputed AAC values. For a given gene, Pearson correlation was applied to assess the similarity of measured expression across studies.

### 2.3 Meta-analysis methods

Let us consider *G* matched genes across *K* studies. For a given study *k* (*k* = 1, … , *K*), denote by *x*_*glk*_ the standardized gene expression intensity of gene *g* (*g* = 1, … , *G*) and cell line *l* (*l* = 1, … , *L*_*k*_), where *L*_*k*_ represents the number of cell lines in study *k*. Let y_*lk*_ be the standardized imputed drug response variable of cell line *l* and study *k*. Under the breast cancer data, a standardized linear regression model was applied to assess the association of gene expression and imputed drug response, while adjusting for the tissue types to evaluate pan-cancer data (Peterson and Brown, 2005). For each individual gene-drug association analysis, estimated effect, standard error of estimated effect, and *p*-value were obtained. To identify a broad inferential basis for evaluation of estimated effects, meta-analysis techniques were applied to integrate results of separate independent studies including combining *p*-values and combining estimated effects (Supplementary Figure S2A).

#### 2.3.1 Combination of *p*-values

Combining the *p*-values from multiple independent studies offers the benefits of simplicity and extensibility to different kinds of outcome variables (Tseng et al., 2012). Fisher’s method (Fisher et al., 1934) and Stouffer’s method (Stouffer et al., 1949) are widely used to combine *p*-value results from different independent studies.

For each gene, Fisher’s method sums up the log transformed *p*-values obtained from individual study (Fisher et al., 1934). The combined Fisher’s statistic $\chi_{\text{Fisher}}^{2}=-2\sum_{k}\log\left(p_{k}\right)$ follows a chi-squared *χ* ^2^ distribution with 2*K* degrees of freedom under the null hypothesis.

For each gene, Stouffer’s method sums the inverse normal transformed *p*-values (Stouffer et al., 1949). The combined Stouffer’s statistic $T_{\text{Stouffer}}=\sum_{k}z_{k}/\sqrt{K}$ ; where *z*_*k*_ = Φ^−1^(*p*_*k*_) and Φ is a standard normal cumulative distribution function; it follows a standard normal distribution under the null hypothesis.

Major limitations of such classical methods are that they do not account for the data heterogeneity and direction of effects. Additionally, such methods are performed under the parametric assumption that *p*-values are uniformly distributed under the null hypothesis, though in practice such an assumption is not always satisfied (Marot et al., 2009).

#### 2.3.2 Combination of estimated effects

Let *β*_*k*_ and *s*_*k*_ be the estimated effect and its standard error in study *k*, respectively. The most popular parametric statistical methods for combining effects are based on fixed- and random-effects (FE & RE) models (Cochran, 1954; Borenstein et al., 2021). The FE model assumes that all studies in meta-analysis share a single true effect *β* (i.e., average effect across all studies) is specified as

$$\beta_{k}=\beta +\epsilon_{k},$$

where *ϵ*_*k*_ is assumed to be independently distributed as $\epsilon_{k}∼N\left(0,\sigma_{k}^{2}\right)$ and within-study variance $\sigma_{k}^{2}$ . Under the FE model, estimated effects are assumed to be homogeneous across studies and all differences in observed effects are due to sampling error or within-study variability, while in practice such an assumption is questionable. One of the challenges in meta-analysis is that true effects are different across studies. Hence, the RE model was proposed to incorporate the variability of the estimated effects across studies in addition to the within-study variability using a two-stage hierarchical process as follows (DerSimonian and Laird, 1986; Brockwell and Gordon, 2001; Viechtbauer, 2005; Bodnar et al., 2017)

$$\beta_{k}=\beta +\eta_{k}+\epsilon_{k},$$

where *η*_*k*_ indicates the error accounting for the between-study variability. Assume error *η*_*k*_ is independently distributed as *η*_*k*_ ∼ *N*(0, *τ* ^2^), where *τ* ^2^ is the between-study or heterogeneity variance of *η*_*k*_ around *β*. The marginal distribution of *β*_*k*_ follows the normal distribution as $\beta_{k}∼N\left(\beta ,\tau^{2}+\sigma_{k}^{2}\right)$ , where *η*_*k*_ and *e*_*k*_ are independent. A common assumption is that each study is based on studies large enough to consider the within-study variance as known and equal to the estimate provided by each study (i.e., $s_{k}^{2}$ ).

The goal is to estimate the overall effect *β*. Following the summarized assumptions in Supplementary Table S1, several approaches have been suggested for combining individual study results into an overall estimate of effect applying an inverse variance scheme (Egger et al., 2008). Under the FE model, the most flexible and widely used inverse variance-weighted average approach was proposed to estimate the overall effect as

$$\hat{\beta }=\frac{\sum_{k=1}^{K}w_{k}\beta_{k}}{\sum_{k=1}^{K}w_{k}},\;\;\;\;\;w_{k}=\frac{1}{s_{k}^{2}}.$$

However, under the RE model, the proposed inverse variance approach is considered to estimate the overall effect where $w_{k}=1/\left(\tau^{2}+s_{k}^{2}\right)$ and the estimate of overall effect depends on the between-study variance. *w*_*k*_ provides the uniformly minimum variance unbiased estimator of *β*. A Wald-type 95% confidence interval is estimated for the overall effect using the normal approximation (DerSimonian and Laird, 1986). The proposed RE meta-analysis approaches are typically performed in two stages, where first the heterogeneity parameter is estimated, and then the effect estimate is derived using the estimated heterogeneity. One of the most troublesome challenging aspects of meta-analysis is the determination of whether there is true heterogeneity, as it can influence the choice of the statistical method to combine estimated effects.

#### 2.3.3 Measures of between-study heterogeneity

Under the RE model, the detection of heterogeneity requires testing the null hypothesis *τ* ^2^ = 0, which corresponds to the FE model. The homogeneity hypothesis is tested using the *Q* statistic (DerSimonian and Kacker, 2007) given by

$$Q=\overset{K}{\underset{k=1}{\sum }}{\left(\beta_{k}-\hat{\beta }\right)}^{2}w_{k}$$

which has an asymptotic chi-squared *χ* ^2^ distribution with *K*−1 degrees of freedom under the hypothesis of consistent or homogeneous association, and $\hat{\beta }$ and *w*_*k*_ are computed using the FE model analysis. The DerSimonian and Laird (DL) estimator DerSimonian and Laird (1986) is possibly the most frequently used approach to estimate the between-study heterogeneity *via* moments approach given by

$${\hat{\tau }}^{2}=\max\left\{0,\frac{Q-\left(K-1\right)}{\sum_{k=1}^{K}w_{k}-\sum_{k=1}^{K}w_{k}^{2}/\sum_{k=1}^{K}w_{k}}\right\}.$$

Note that the *Q* test suffers from low power when studies have small sample size (*L*_*k*_) or are few in number (*K*) (Hoaglin, 2016). More importantly, the *Q* statistic and the estimate of between-study variance depend on the scale of effects. Hence, neither *Q* nor ${\hat{\tau }}^{2}$ can be used to compare degrees of heterogeneity between different meta-analyses (Higgins et al., 2003). Several approaches have been suggested over the years for how best to assess homogeneity across studies, and the corresponding research is ongoing.

The relative amount of between-study heterogeneity can be expressed in terms of the measure of $I^{2}=\tau^{2}/\left(\tau^{2}+{\tilde{\text{s}}}^{2}\right)$ that reflects the proportion of total variance that is attributed to heterogeneity (Higgins et al., 2003). ${\tilde{\text{s}}}^{2}$ is required as an estimate of the within-study variances. Unlike the *Q* test, *I* ^2^ can be directly compared between meta-analyses with different numbers of studies and different types of outcome data and also does not inherently depend on the number of studies in the meta-analysis (Higgins et al., 2003). Due to the low power situation of meta-analyses, Higgins et al. (2003) suggested that we could tentatively assign a significant degree of heterogeneity (i.e., substantial heterogeneity) when *I* ^2^ > 50% and *Q* test *p*-value <0.1 (Hoaglin, 2016).

Many alternatives to the DL approach have been proposed such as modifications of the method of moments, likelihood principle, model error variance, Bayes, and the non-parametric approaches with the aim of avoiding distributional assumptions (Veroniki et al., 2016; Guolo and Varin, 2017; Langan et al., 2019). In this study, we focus on six different heterogeneity variance estimators including moments estimators such as DerSimonian and Laird (DL) (DerSimonian and Laird, 1986), Paule and Mandel (PM) (Paule and Mandel, 1982), Hedges (HE) (Hedges and Olkin, 2014) and Hunter-Schmidt (HS) (Schmidt and Hunter, 2004), error variance estimator Sidik-Jonkman (SJ) (Sidik and Jonkman, 2007), and empirical Bayes (EB) estimator (Sutton and Abrams, 2001; Turner et al., 2012). A common problem with such estimators is that they frequently produce higher numbers for small meta-analyses, and lower for analyses involving many studies which leads to inadequate heterogeneity and effect estimates along with challenges in the choice of FE or RE analysis methods (Turner et al., 2012; Bodnar et al., 2017; Langan et al., 2019).

To compare meta-analyses results, within each study, the effect size for each gene was measured. Meta-analyses for each gene were performed across studies using the *meta* (Schwarzer et al., 2019) and *metap* (Dewey, 2018) R packages. To correct for multiple testing, the Benjamini & Hochberg procedure was used to control false discovery rate (FDR) (Benjamini and Hochberg, 1995). The Jaccard coefficient index was applied to compare the similarity between the identified top-ranked genes associated with a drug using different independent meta-analyses. An UpSet plot was applied to visualize the number and the overlap of genes associated with drugs by considering different meta-analysis methods and volcano plot to highlight statistically significant gene-drug associations.

#### 2.3.4 Bayesian approaches

Bayesian inference has been suggested in the context of meta-analysis to capture uncertainty in estimation of the heterogeneity by incorporating prior information (Sutton and Abrams, 2001; Bodnar et al., 2017; Röver, 2017). Compared to the earlier discussed approaches, Bayesian methods offer several potential advantages which include producing a distribution for the effect and heterogeneity, leading to credible intervals for overall effect and heterogeneity.

Within the explained two-stage hierarchical model (Supplementary Table S1), to infer the unknown hyper parameters *β* and *τ*, prior knowledge needs to be specified where the joint prior probability can be factored into independent marginals *p*(*β*, *τ*) = *p*(*β*) × *p*(*τ*). The range of reasonably specified priors is usually limited. Non-informative or weakly informative priors for the effect *β* are usually applied. However, informative normal prior constitutes the conditionally conjugate prior distribution for the effect along with being computationally convenient (Röver, 2017). In addition, for the between-study heterogeneity *τ* an informative prior is often appropriate especially when only a small number of studies is involved (Bodnar et al., 2017; Röver, 2017). Let

$$p\left(\beta ,\tau \right)\propto \tau \sqrt{\overset{K}{\underset{k=1}{\sum }}{\left(\frac{1}{\sigma_{k}^{2}+\tau^{2}}\right)}^{2}},$$

denote the non-informative uniform and Jeffreys priors for parameters *β* and *τ*, respectively Bodnar et al. (2017); Röver (2017). The Jeffreys prior’s dependence on the standard errors *σ*_*k*_ implies that the prior information varies with the precision of the underlying data *β*_*k*_. Posterior density may be accessed in quasi-analytical form and 95% central credible intervals derived from a posterior probability distribution can be constructed using the *bayesmeta* R package Röver (2017). We draw attention to this Bayesian procedure by comparing its performance with commonly used classical DL meta-analysis procedure.

### 2.4 Assessing the impact of duplication of data across studies

Duplication in study effects has been defined as the estimated effect results from a complete replication of a particular study or from some subset of measured data (Wood, 2008; Cheung, 2019). The dependency in the meta-analysis is studies at the effects level, which can then be caused by duplication of input data (e.g., gene expression), output data (e.g., drug response measurements) or a combination of both (Supplementary Figure S2B).

Aggregating non-independent effects will bias the estimated overall effect in meta-analyses Wood (2008). For a given study *k*′ (*k*′ = 1, … , *K*), let $x_{glk^{\prime }}^{⋆}$ be the missing gene expression intensity of gene g across cell lines *l* = 1, … , *L*, where L matched cell lines is considered across studies (i.e., *L*_*k*_ = *L*, *∀k* = 1, … *K*). Assume available measured expression data *x*_*glk*_ such that *k*′ ≠ *k*, *∀k* = 1, … , *K* be duplicated for the missing data (i.e., $x_{\mathrm{glk}}\to x_{glk^{\prime }}^{⋆}$ ) which leads to a lack of independence of effects across studies (i.e., *β*_*k*_ ⊥⊥̸ *β*_*k*′_). In each meta-analysis, Γ(*γ*, *K*) denotes a set of all possible dependent studies by computing *γ*-combination of K studies, where *γ* = 1, … , *K*−1 is the number of missing expression data.

One of the considerable consequences of traditional meta-analyses of non-independent studies compared with independent ones is the significant differences in the estimate of overall effect (Wood, 2008). Hence, for a given gene and *γ*, to investigate the bias of ignoring the independence assumption of effects, the mean absolute deviation (MAD) metric is computed as

$$\text{MAD}=\frac{1}{\left|\Gamma \right|}\underset{d\in \Gamma }{\sum }\left|{\hat{\beta }}_{d}-\hat{\beta }\right|,$$

where $\hat{\beta }$ and ${\hat{\beta }}_{d}$ demonstrate the estimated overall effect by using the independent and non-independent studies, respectively. In addition, we evaluate the association between increases in the number of non-independent studies and the similarity of measured expression across studies. The Jaccard similarity index was applied to compare the similarity between the detected top-ranked genes associated with a drug over duplicated study effects. We evaluate whether there is an increasing trend in bias over the number of non-independent studies. Trend detection non-parametric Mann-Kendall (MK) (Mann, 1945; Pohlert, 2016) method is considered to test the null hypothesis *H*_0_: there is no monotonic trend in bias, *versus* the alternative hypothesis *H*_*a*_ of an increasing trend of bias when the number of studies increase. The MK trend test follows asymptotically a normal distribution where a lack of samples may decrease the power of analysis to detect trends.

### 2.5 Simulation study design

Most simulation studies currently explore the performance of various methods by focusing on measuring inconsistency in meta-analyses rather than in dealing with dependent effects across studies. Therefore, we conducted a simulation study to investigate the performance of the widely used RE meta-analysis approach (DL) in estimating the overall effect in which the dependence of the effects are ignored. We explored the effects of different parameters, varied in the simulations including the heterogeneity across studies (*τ* ^2^), number of studies (*K*), variation in studies $\left(\sigma_{k}^{2}\right)$ , correlation between random effects (*ρ*), and overall effects (*β*). When effects were correlated across studies, the traditional RE method (DL) was applied to assess whether and for which conditions lack of independence assumption across studies produced significantly different results. In this study, we focused on the overall effect estimate and on its standard error, because applied researchers are often mostly interested in testing overall effect.

#### 2.5.1 Simulation method and performance measures

Parameter estimates and their performance measures are assessed in a 3(*K*) × 4 (*τ* ^2^) × 3 (*σ* ^2^) × 3(*β*) × 4(*ρ*) factorial design (Table S2), where number of studies, heterogeneity across studies, variation within-studies, overall effects, and correlation between random effects are varied and each scenario is tested using a DL meta-analysis approach. Note that the within-study variance is assumed to be equal. Under the explained two-stage hierarchical model, for studies 1, … , *K* in each meta-analysis, true effects (*β*_1_, … , *β*_*K*_)′ are simulated from multivariate normal distribution *N*_*K*_(*β* ***I***_*K*_, Σ), where ***I***_*K*_ is a *K* × *K* identity matrix and the symmetric variance-covariance matrix

$$\Sigma =\left[\begin{array}{ccccc} \tau^{2}+\sigma_{1}^{2} & \rho_{12}\sigma_{1}\sigma_{2} & \rho_{13}\sigma_{1}\sigma_{3} & \cdots & \rho_{1k}\sigma_{1}\sigma_{K}\\ \rho_{21}\sigma_{2}\sigma_{1} & \tau^{2}+\sigma_{2}^{2} & \rho_{23}\sigma_{2}\sigma_{3} & \cdots & \rho_{2K}\sigma_{2}\sigma_{K}\\ \vdots & \vdots & \vdots & \ddots & \vdots \\ \rho_{K1}\sigma_{K}\sigma_{1} & \rho_{K2}\sigma_{K}\sigma_{2} & \rho_{K3}\sigma_{K}\sigma_{3} & & \tau^{2}+\sigma_{K}^{2} \end{array}\right]$$

Parameter values are chosen to represent the range of values observed in the pharmacogenomic meta-analysis. The number of studies to be combined is manipulated at 3 levels (3, 7, and 10) to simulate a statistical combination of a range of meta-analytic preclinical pharmacogenomic studies. The Variation within studies represents that large number of cell lines within studies produces smaller within-study variance, which influences overall effect estimation.

Heterogeneity variance parameter values (*τ* ^2^) are defined such that the resulting meta-analyses vary through a wide range of levels of inconsistency between study effects. A between-study variance of 0.001 would signify that almost no true heterogeneity exists. The correlation selected to calculate the covariances between study effects ranges between 0.1 and 0.7. To set one duplication scenario, the *ρ*_12_ had values 0.1 and 0.7, and to increase the number of duplications to two and three, *ρ*_23_ and *ρ*_13_ had values 0.2 and 0.6, respectively. Finally, the overall effects are chosen to be 0.2, 0.5, and 0.8 which are representing small, medium, and large gene-drug correlation association.

Simulating all combinations of parameter values leads to 432 scenarios for non-independent effects meta-analyses. The overall effect estimates were summarized across the 1,000 iterations. To evaluate the properties of the effect estimators, we estimated the relative mean squared error (MSE) and the coverage probability of the 95% confidence intervals by recording the percentage of replications where intervals included the true overall effect.

## 3 Results

### 3.1 Comparison of meta-analysis techniques: Independent studies

We performed gene-drug association analysis for each study using (adjusted) standardized linear regression model to obtain estimated effect and its standard error along with *p*-value per gene. Meta-analysis approaches were applied to integrate information across studies including Fisher and Stouffer methods to combine *p*-values and the RE model to integrate estimated effects across individual studies. Meta-analysis approaches differ on how they treat heterogeneity. Hence, various heterogeneity approaches such as DL, HS, PM, HE, SJ, and EB were compared (see Materials and Methods section).

To assess the performance of different meta-analysis approaches used to identify significant gene-drug associations using breast cancer and pan-cancer pharmacogenomic datasets, we compared the lists of genes significantly associated with drug response (FDR <0.05; Supplementary Figure S3). The *p*-value combination methods (Fisher and Stouffer) are almost the most conservative (least number of significant genes) meta-analyses. SJ is typically less conservative compared to *p*-value combining methods, while it is most conservative among the RE meta-analysis approaches. HS is always least conservative (greatest number of significant associated genes). In addition, a large percentage (∼80−97%) of genes are found in common across the methods that are not most conservative. Other methods including HE, EB and PM demonstrate the similar pattern to DL method with more than 80% of commonly identified significant associated genes.

To compare the performance of different meta-analyses, we computed the Jaccard index to assess the similarity between the ranked lists of gene pairs obtained from different meta-analyses (Supplementary Figure S4). We find that the top 100 ranked gene-pairs are most similar with a range from 65% to 100% across combining effects approaches except for the error variance estimator SJ. This observation of similarity measures suggests that meta-analyses are not identical and there is considerable diversity between combining *p*-values and effects approaches. Different types of meta approaches had been reviewed and compared (Tseng et al., 2012; Sweeney et al., 2017). Hence, we chose the less conservative and most commonly used DL method to estimate heterogeneity for the rest of the meta-analyses.

### 3.2 Breast cancer biomarker discovery

**FIGURE 2:**
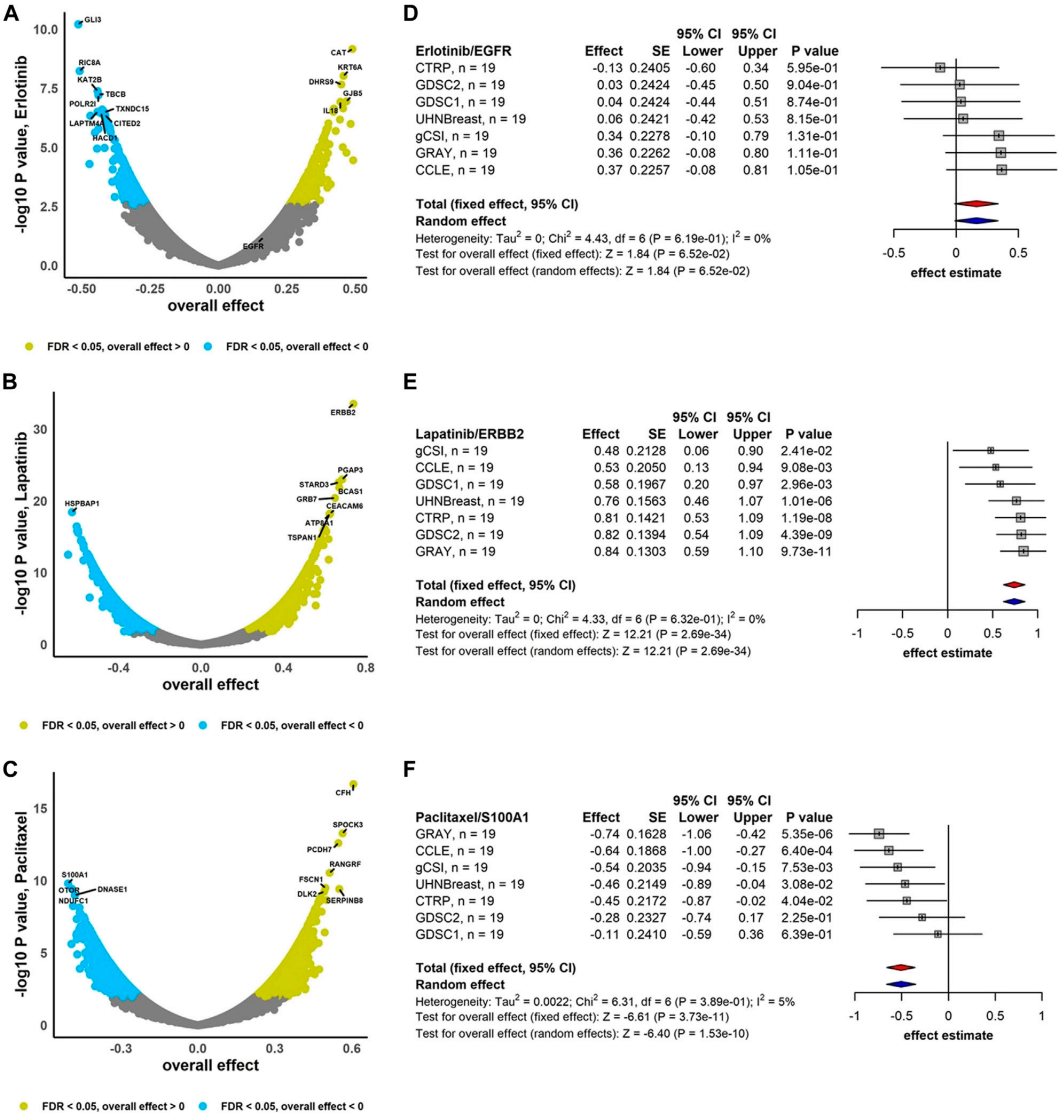
Breast cancer independent meta-analyses. Volcano plots show genes associated with drug response using RE meta-analysis model and forest plots illustrate overall effect estimate using FE (red diamond) and RE (blue diamond) meta-analysis models using drugs **(A)** Erlotinib, **(B)** Lapatinib, and **(C)** Paclitaxel. DL approach was applied to estimate heterogeneity across studies.

We performed the association analysis between genes and drug response (AAC) using the RE meta-analysis model including the DL heterogeneity estimation approach to combine estimated effects (Pearson correlation coefficient) across breast cancer studies (Figure 2). Drugs Erlotinib and Lapatinib demonstrate that 60% and 62% of genes are negatively correlated with FDR <0.05, respectively, i.e., higher gene expression is associated with lower drug response, and therefore lower drug activity (Figures 2A,B). However, Paclitaxel with 59% of genes correlated positively with FDR <0.05 represents higher gene expression is associated with higher drug activity (Figure 2C). In addition, ERBB2 is highly sensitive to Lapatinib (overall effect = 0.74, 95% CI: 0.62 to 0.86, *p* = 2.69e-34), while Paclitaxel and S100A1 are negatively associated (overall effect = -0.51, 95% CI: −0.66 to -0.35, *p* = 1.53e-10) (Figure 2). Moreover, EGFR demonstrates less sensitivity to Erlotinib (overall effect = 0.16, 95% CI: −0.01 to 0.34, *p* = 6.52e-02) compared to the breast cancer meta-analyses where not limited to the common cell lines (overall effect = 0.26, 95% CI: 0.16 to 0.36, *p* = 2.47e-07) (Supplementary Figure S5). For breast cancer gene-drug association meta-analyses, around 8%–13% of genes have substantial estimated heterogeneity, where no greater than 1% of them are significantly associated with drugs. However, almost 10%–30% of genes are associated to drugs with non-substantial estimated heterogeneity (Supplementary Figure S6A).

### 3.3 Pan-cancer biomarker discovery

**FIGURE 3:**
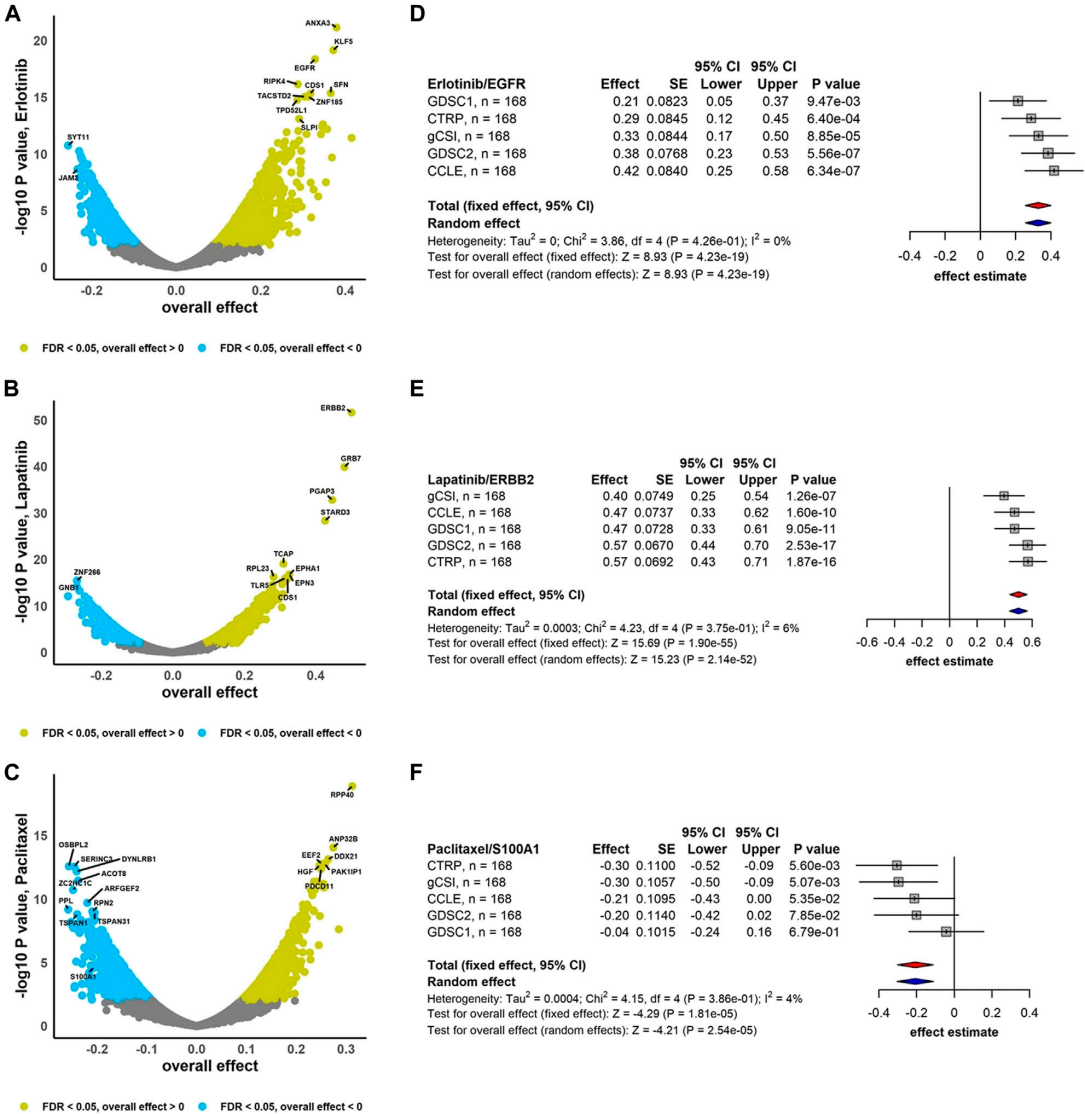
Pan-cancer independent meta-analyses. Volcano plots show genes associated with drug response using RE meta-analysis model and forest plots illustrate overall effects estimate using FE (red diamond) and RE (blue diamond) meta-analysis models using drugs **(A)** Erlotinib, **(B)** Lapatinib, and **(C)** Paclitaxel. DL approach was applied to estimate heterogeneity across studies.

We considered the integration of estimated effects to assess the gene-drug association across pan-cancer data using RE meta-analysis including the DL heterogeneity estimation approach (Figure 3). We obtained that 50% and 60% of genes are negatively associated (FDR <0.05) with Erlotinib and Lapatinib, respectively, i.e., higher gene expression was associated with lower drug activity, while 51% of genes are positively associated with drug Paclitaxel (Figure 3). EGFR and ERBB2 show considerable sensitivity to Erlotinib (overall effect = 0.33, 95% CI: 0.26 to 0.40, *p* = 4.23e-19) and Lapatinib (overall effect = 0.50, 95% CI: 0.44 to 0.56, *p* = 2.14e-52), respectively (Figure 3). However, S100A1 is significantly resistant to Paclitaxel (overall effect = -0.21, 95% CI: -0.30 to -0.11, *p* = 2.54e-05) (Figure 3). Compared to the breast cancer meta-analysis, the pan-cancer gene-drug association meta-analyses contain less studies and more cell lines where almost 5%–13% of genes have substantial estimated heterogeneity and around 1% or less of them are associated with drugs. Moreover, significant gene-drug associated with non-substantial estimated heterogeneity ranges between 16%–20% (Supplementary Figure S6B).

### 3.4 Comparison of frequentist and bayesian meta-analysis

To capture uncertainty in estimation of the heterogeneity, we applied Bayesian technique by incorporation Jeffreys prior information. We compared the performance of DL and Bayesian approaches to estimate heterogeneity and overall effect along with 95% confidence or credible regions. DL and Bayesian procedures yield almost the same estimates for the overall effect (Supplementary Figure S7). For the breast cancer meta-analyses, the Bayesian credible interval is between 1.37 and 1.45 times wider than the DL interval, while as the number of studies decreases using pan-cancer meta-analyses, the Bayesian interval gets wider from 1.66 to 1.68 times of DL interval. The conventional DL method detects no or low heterogeneity, while Bayesian estimate of *I* ^2^ ranges between 38% and 56%. In addition, across all genes, to compare the impact of Bayesian and DL methods, the length of 95% confidence and credible regions along with estimate of *I* ^2^ were computed (Supplementary Figures S8, S9). The median of length of Bayesian intervals is 1.33–1.40 times of DL interval using breast cancer data, while considering pan-cancer data, the median of length of Bayesian credible becomes around 1.52 to 1.54 times of DL interval across all drugs (Supplementary Figures S8, S9A). The ranges of median of *I* ^2^ Bayesian estimates are (36%–42%) and (47%–53%) using breast cancer and pan-cancer meta-analyses, respectively (Supplementary Figures S8, S9B). Our results are comparable to those reported by Bodnar et al. (2017) due to assuming the same priors.

### 3.5 Simulation study and non-independent effects

**FIGURE 4:**
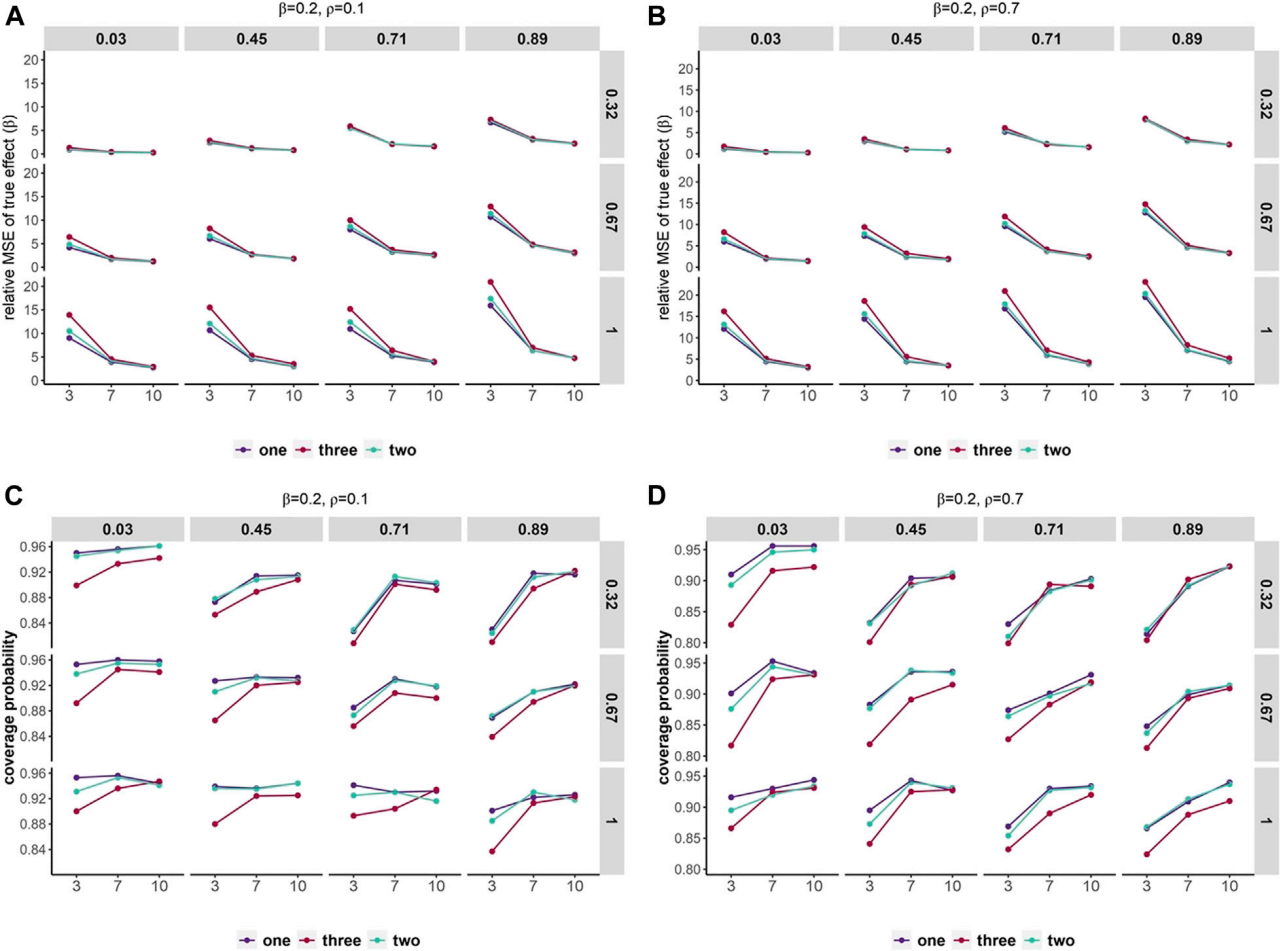
Mean squared error and coverage probability of overall effect estimates. Scenarios containing various within-study variances (row) and heterogeneity across studies (column). The *x*-axis represents the number of studies and the *y*-axis shows the relative MSE **(A,B)** and coverage probability of 95% confidence intervals **(C,D)**. Set overall effect *β* = 0.2. Different colors represent a number of duplication or non-independent effects across studies.

In traditional DL meta-analysis where the independence of effects was ignored, higher relative MSEs were observed as the number of non-independent effects increased (Figure 4, Supplementary Figures S10, S11). However, relative MSEs were similar across different numbers of non-independent effects in scenarios with small variation within and across studies and large numbers of studies. In scenarios with a small number of studies, relative MSEs were higher than scenarios with larger numbers of studies. In addition, as the variation within and across studies increased, the relative MSEs gradually rose. We also observed that large correlation across the effects can yield slightly higher relative MSE. As the overall effect increased, the relative MSEs had considerable decreases across all scenarios.

Coverage of the 95% confidence interval can differ by 5%–15% in all scenarios (Figure 4, Supplementary Figures S10, S11). As the number of non-independent effects increased, lower coverage was observed. Coverage had variations up to 10% between numbers of studies. Coverage varies between almost 90% and 96% when studies are homogeneous and up to two non-independent effects included and can be as low as 80% in scenarios with a small number of studies and three non-independent effects. In addition, large correlation across the effects can yield slightly lower coverage than smaller correlation. As the overall effect increased, the coverage decreased gradually in all scenarios.

The summary results indicated that the coverage of the 95% confidence intervals improved (or relative MSE decreased) as the number of studies increased, the variation within and across studies decreased, and had a smaller number of duplications across studies.

### 3.6 Duplicate study effects in a meta-analysis: Application in pharmacogenimic datasets

Duplication in study effects has been defined when estimated effect results from a complete replication of a particular study or from some subset of measured data. For instance, the generated expression duplicated in the study contains missing expression data for a given gene (Supplementary Figure S2B). To assess the bias of ignoring the dependence of the effects, we considered matched cell lines across non-independent studies for breast cancer and pan-cancer meta-analyses individually. Therefore, across whole genes, we considered all possible duplicated study effects and applied the RE model, including DL heterogeneity estimation approach to integrate the duplicate study effects, to estimate overall effect and heterogeneity. Reflecting on duplicate data problems, conclusions based on the traditional meta-analyses will bias the aggregated estimated effects and can increase the false positive results. Additionally, bias is determined using the mean distance between the estimated overall effect using non-independent studies and the overall effect by integrating independent studies (i.e., MAD). Additionally, we investigate the association of violating the independence assumption over the similarity of measured expression per gene across studies by applying the Pearson correlation. The median of estimated Pearson correlation can be classified as low |*r*| < 0.3, medium 0.3 ≤ |*r*| < 0.7 and high |*r*|≥ 0.7 (Mukaka, 2012).

#### 3.6.1 Bias assessment in non-independent meta-analysis: Breast cancer and pan-cancer data

**FIGURE 5:**
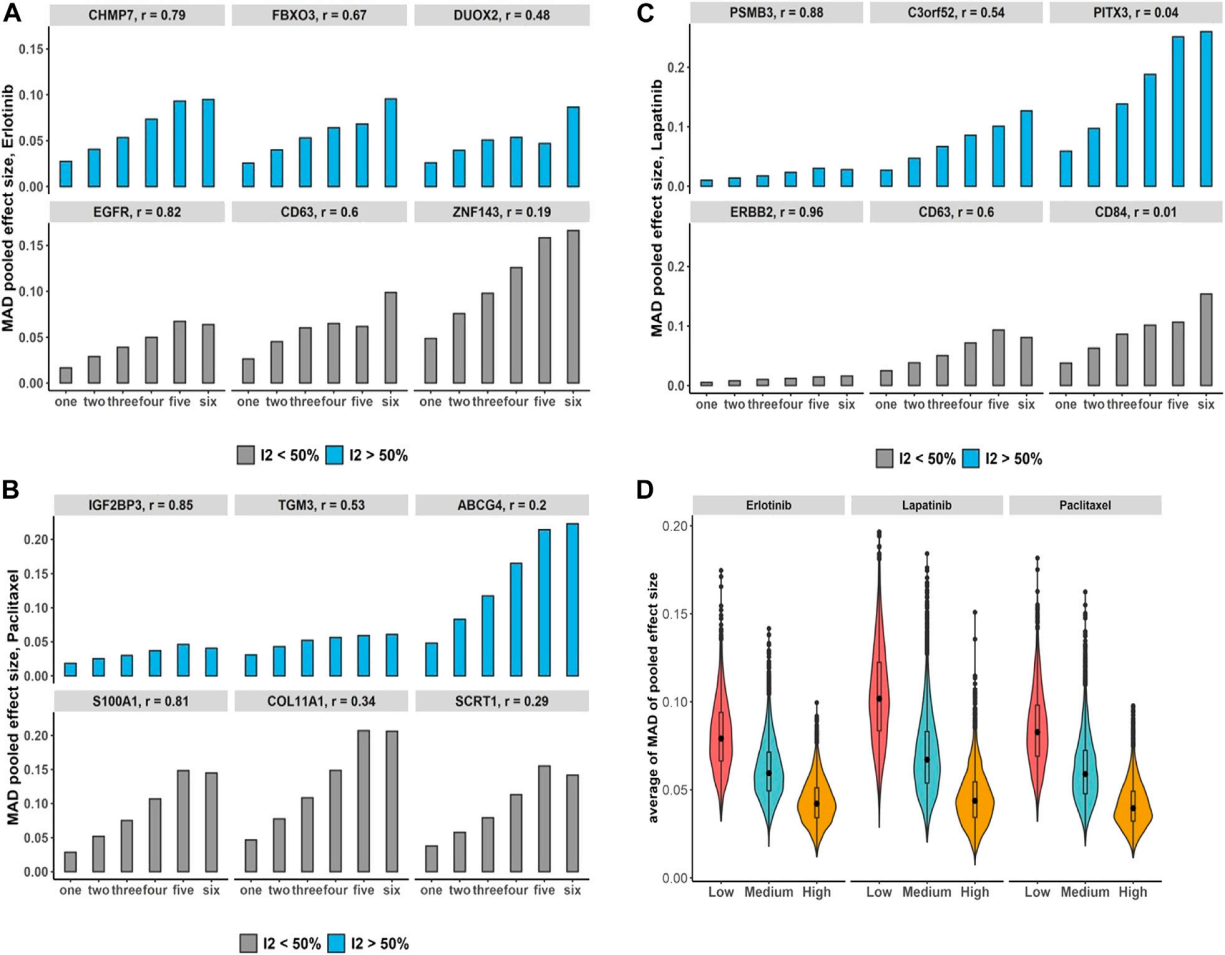
Breast cancer non-independent meta-analyses: **(A–C)** bar plots demonstrate increases in the number of duplications and its impact on the bias of estimated overall effect using MAD metric across drugs and selected genes with substantial (blue) and non-substantial (gray) estimated heterogeneity estimation. Note that *x*-axis presents the number of duplicate study effects. **(D)** Violin plots show average of MAD values across non-independent analyses per genes over median of Pearson correlation of each gene’s expression across studies: low (|*r*| < 0.3), medium (0.3 ≤ |*r*| < 0.7), and high (|*r*|≥ 0.7). Black dot at the box plot represents the median.

We assessed the increases in the number of duplication using breast cancer data and its impact on the bias of the estimated overall effect using the MAD across drugs and selected genes with estimated substantial and non-substantial heterogeneity (Figures 5A–C). The results indicate that increases in the number of duplicate study effects can considerably raise the bias of meta-estimates of effects. In addition, testing the trend of bias across all genes by growth in the number of non-independent studies denotes almost 97%–99% of genes following an increasing trend with *p*-value <0.05 across drugs. Moreover, we obtain genes with higher median Pearson correlations that show on average less bias compared with genes that have low levels of median correlations across studies (Figure 5D).

**FIGURE 6:**
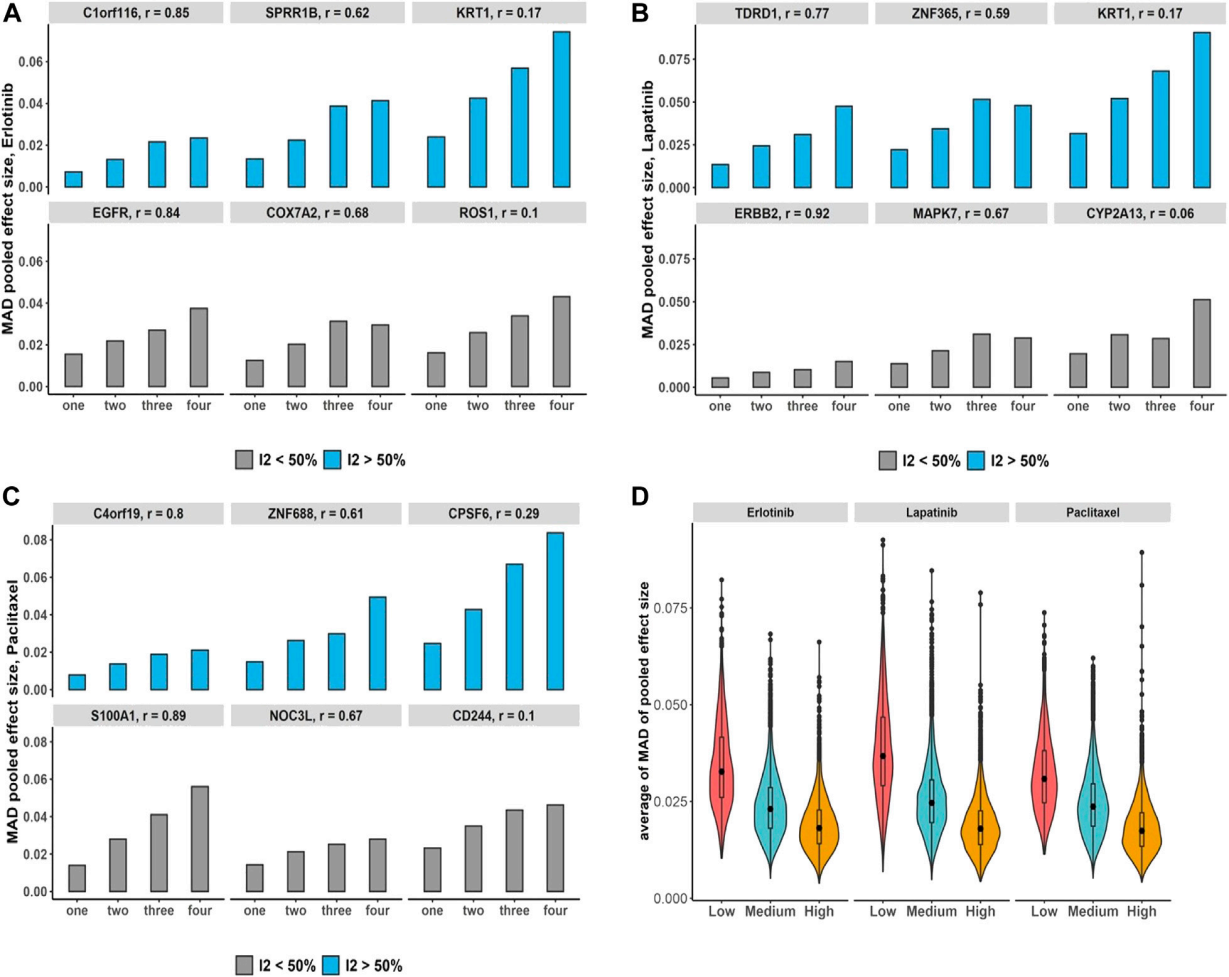
Pan-cancer non-independent meta-analyses: **(A–C)** bar plots demonstrate increases in the number of duplications and its impact on the bias of estimated overall effect using MAD metric across drugs and selected genes with substantial (blue) and non-substantial (gray) estimated heterogeneity estimation. Note that *x*-axis presents the number of duplicate study effects. **(D)** Violin plots show average of MAD values across non-independent meta-analyses per genes over median of Pearson correlation of each gene’s expression across studies: low (|*r*| < 0.3), medium (0.3 ≤ |*r*| < 0.7), and high (|*r*|≥ 0.7). Black dot at the box plot represents the median.

Additionally, under the pan-cancer non-independent meta-analyses with more matched cell lines but fewer studies compared with breast cancer data, we obtain a similar increasing pattern in computed bias (Figures 6A–C). To evaluate the bias changes across whole genes when more duplicate study effects were added in meta-analyses, MK trend test results show almost 86%–93% of genes with *p*-value <0.05 were detected across drugs. Moreover, similar to the breast cancer data, the average of bias across whole genes was reduced as the median of Pearson correlations increases (Figure 6D).

#### 3.6.2 Non-independent meta-analysis and biomarker discovery

**FIGURE 7:**
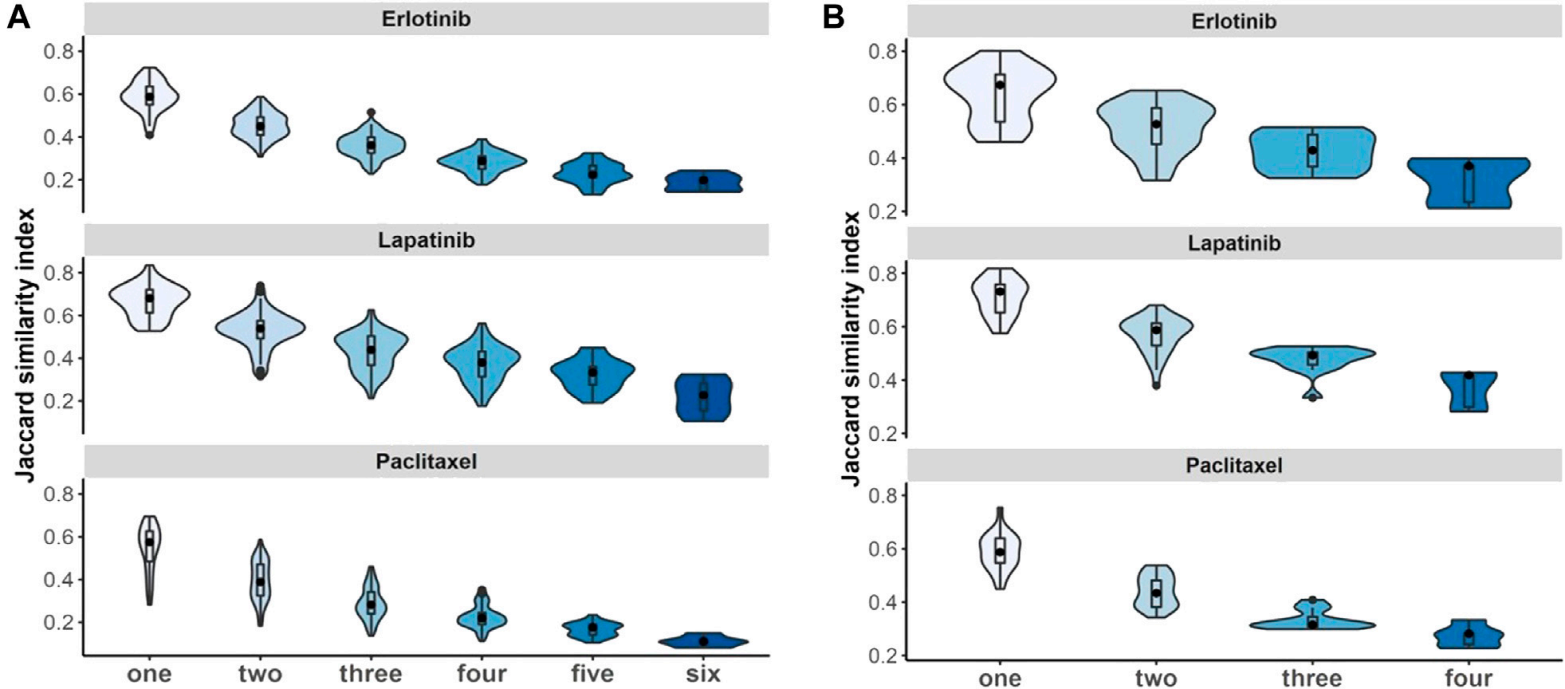
Non-independent studies meta-analyses and biomarker discovery. Violin plots illustrate stability of identified 100 top-ranked genes associated with drugs by increasing the number of duplications using Jaccard similarity index using **(A)** breast cancer and **(B)** pan-cancer data. Black dot at the box plot represents the median. Note that the *x*-axis presents the number of duplicate study effects.

We evaluated whether violating the independence assumption in meta-analyses can impact on the differential expression analyses by considering Jaccard similarity index to compare the 100 top-ranked genes associated with drugs under non-independent and independent meta-analyses. The results illustrate that if we have more duplicate study effects, the overlap of detected expressed genes decreases and leads to a decrease in power of analyses (Figure 7).

#### 3.6.3 Non-independent bayesian meta-analysis

We also investigated how duplicate study effects can bias the meta-analyses using the Jeffreys Bayesian approach and compared the results with traditional DL meta-analysis. Because Bayesian approaches take more time and are computationally expensive, we only considered specific genes used by DL meta-analysis instead of including whole genes. We evaluated the pattern of changes in the number of duplicate study effects and the bias of the estimated overall effect using the Bayesian approach across drugs and selected genes. This process also considered estimated substantial and non-substantial estimated heterogeneity using breast cancer and pan-cancer data, respectively (Supplementary Figures S12, S13A–C). By increasing the number of duplicate study effects, bias of estimated overall effect will increase and genes with higher correlation across studies will indicate less bias. In addition, we observed no considerable trend for the average of 95% intervals using Bayesian and DL meta-analyses by increasing the number of duplicate study effects (Supplementary Figures S12, S13D). The results show that the median length of Bayesian intervals ranges almost between 2.4 and 2.5 times the DL interval using breast cancer data. When considering the pan-cancer data, the median length of Bayesian interval becomes around 4.6 to 4.8 times of DL interval across all drugs.

## 4 Discussion

In this study, we reviewed and compared the performance of various traditional meta-analysis including frequentist and Bayesian approaches to improve the reproducibility of the identification of biomarkers using independent large-scale pharmacogenomic datasets. We observed that meta-analyses are not identical and there is considerable diversity between combining *p*-values and effect sizes approaches. We further assessed the bias of including non-independent effects (or duplicate data) in a conventional meta-analysis. When effects are not independent, conclusions based on these conventional procedures will bias overall effect estimates and inflate the type I error rates (Becker, 2000; Wood, 2008; Lin and Sullivan, 2009; Hedges et al., 2010; Scammacca et al., 2014; Van den Noortgate et al., 2015; Cheung, 2019; Cooper et al., 2019; Liu et al., 2019; Wilson, 2019; Liu and Xie, 2020; Luo et al., 2020; Borenstein et al., 2021). We demonstrated how increases in the number of duplicated studies can impact the bias of overall estimate of effect and the identification of gene-drug associations. We also evaluated whether violating the independence assumption in meta-analyses can impact on the biomarker discovery.

Combining *p*-values and effects approaches are used to aggregate results from separate independent analyses. Although meta-analyses that combine *p*-values have widely been used before, they were not able to address the direction of effects and data heterogeneity (Marot et al., 2009). It was denoted that *p*-values combination methods (Fisher and Stouffer) are the most conservative and the HS RE model is the least conservative to identify genes associated with drugs. Among approaches for estimating between-study variance, SJ RE method is the most conservative method. Assessing heterogeneity is a critical issue in meta-analyses. Several statistical methods are routinely used to identify the statistical significance of heterogeneity which may lead to different estimates of overall effect and different standard errors (Veroniki et al., 2016; Guolo and Varin, 2017; Langan et al., 2019). To address the uncertainty in the estimation of the heterogeneity in a RE model when studies have either small cell lines or are few in number, the Bayesian RE model was proposed (Bodnar et al., 2017). The results indicated that the 95% Bayesian confidence interval is considerably wider than the DL method, while both DL and Bayesian procedures yielded the same estimate for the overall effect. In addition, compared to the Bayesian approach, the DL method detected no heterogeneity which is comparable with reported results by Bodnar et al. (2017).

The most substantial outcomes of the non-independent studies are the significant differences in the estimates of the overall effect. From the results of the simulation study, as the number of non-independent effects increased, higher relative MSE and lower-than-95% coverage probability were observed. However, almost equal relative MSEs across different numbers of non-independent effects were observed in scenarios with small variation within and across studies as well as large numbers of studies. Regarding the pharmacogenomics data analyses, the results indicated that by increasing the number of duplicate study effects, bias of overall effect will increase and genes with higher correlation denote less bias. In addition, when we have more duplicate study effects, the overlap of detected associated genes with drugs decreases and produces low power of analyses and more false positive findings.

Early meta-analytic researchers noted the non-independent study problem and suggested solutions. Doing nothing to correct dependent effects or accepting duplication inflate the Type I error and bias the estimated overall effect. Avoiding dependence by averaging effects (Wood, 2008) or shifting unit-of-analysis (Cooper et al., 2019) can reduce the variance between effects while informative differences get lost. Additionally, including the correlation between effects from the same study using multivariate approach, determining the covariance of effects across studies for overlap samples, and estimating the variance components (e.g., between-studies and within studies) were proposed to deal with non-independent effects in meta-analyses (Becker, 2000; Lin and Sullivan, 2009; Hedges et al., 2010; Van den Noortgate et al., 2015; Luo et al., 2020). The most complex strategy is to model the dependence by proposing multilevel models where the correlation between effect sizes from the same study is not needed and separate estimates of the different variance components are estimated. However, when using multilevel models, it is very important to correctly specify all relevant random effects in the model (Cheung, 2014; Van den Noortgate et al., 2015; Cheung, 2019). Moreover, to combine *p*-values under arbitrary dependency structures, the Cauchy combination test (Liu et al., 2019; Liu and Xie, 2020) and the harmonic mean *p*-value (Wilson, 2019) were proposed. However, the proposed methods may be less powerful or even powerless under some conditions (Chen, 2022).

In conclusion, we should carefully define the inclusion and exclusion criteria and use these criteria to determine whether or not the studies or the effects should be included. We have to properly incorporate the dependence in a meta-analysis by including the covariance of non-independent effects or modeling the dependency. This research area remains challenging. Novel powerful and robust tests for combining non-independent effects are still highly desired.

## Data Availability

Publicly available datasets were analyzed in this study. This data can be found here: https://doi.org/10.24433/CO.2186077.v1.
