# Supplementary material for: The impact of violating the independence assumption in meta-analysis on biomarker discovery
Source: Front Genet. 2023 Jan 4;13:1027345. doi: 10.3389/fgene.2022.1027345 (PMC9885264; doi:10.3389/fgene.2022.1027345)
Supplement: Supplementary file 1 [file DataSheet1.pdf]

Supplementary Material

1 SUPPLEMENTARY TABLES AND FIGURES

Fixed-effects Model	Random-effects Model
$\beta_k = \beta + e_k$	$\beta_k = \beta + \eta_k + e_k$
$e_k \sim N(0, \sigma_k^2)$	$\eta_k \sim N(0, \tau^2)$
$\beta_k \sim N(\beta, \sigma_k^2)$	$e_k \sim N(0, s_k^2)$
$w_k = \frac{1}{\sigma_k^2}$	$\beta_k \mid \eta_k \sim N(\mu, \tau^2 + s_k^2)$
$\hat{\sigma}_k^2 = s_k^2$ (large L_k)	$w_k = \frac{1}{\tau^2 + s_k^2}$
	η_k and e_k are independent

Table S1. Models to incorporate study results in a meta-analysis.

Factor (number of conditions)	Simulation conditions
number of studies (3)	$K = 3, 7, 10$
Within-study variance (3)	$\sigma^2 = 0.1, 0.45, 1$
Between-studies variances (4)	$\tau^2 = 0.001, 0.2, 0.5, 0.8$
Correlation between study random effects (4)	$\rho_{12} = 0.1, 0.7 \quad \rho_{23} = 0.2 \quad \rho_{13} = 0.6$
Overall effects (3)	$\beta = 0.2, 0.5, 0.8$

Table S2. Summary of the simulated factor conditions. The within-study variance for each study is assumed to be equal.

SUPPLEMENTARY FIGURES

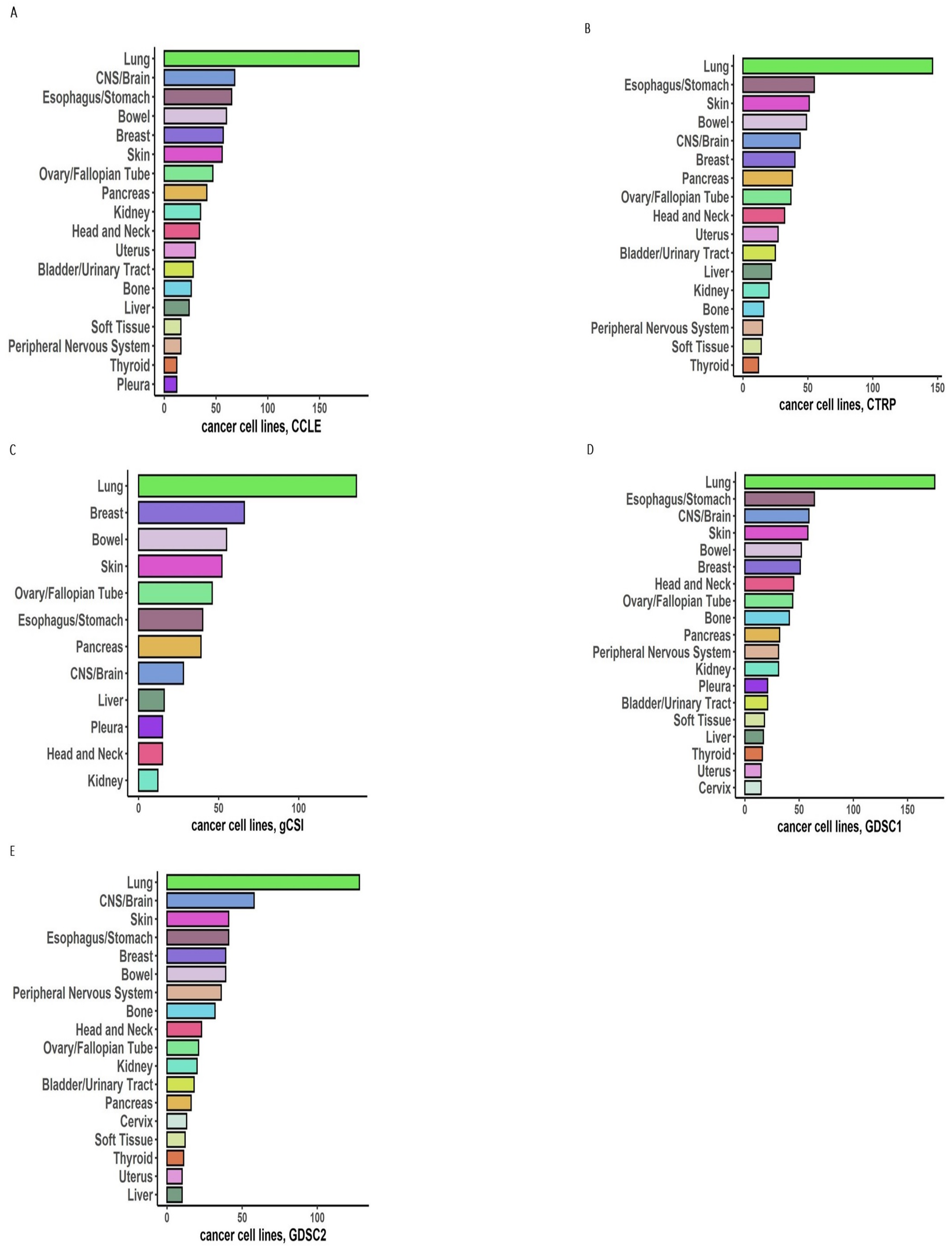

Figure S1. Pan-cancer cell line data. Bar plots illustrate distribution of cell lines across studies and tissue types.

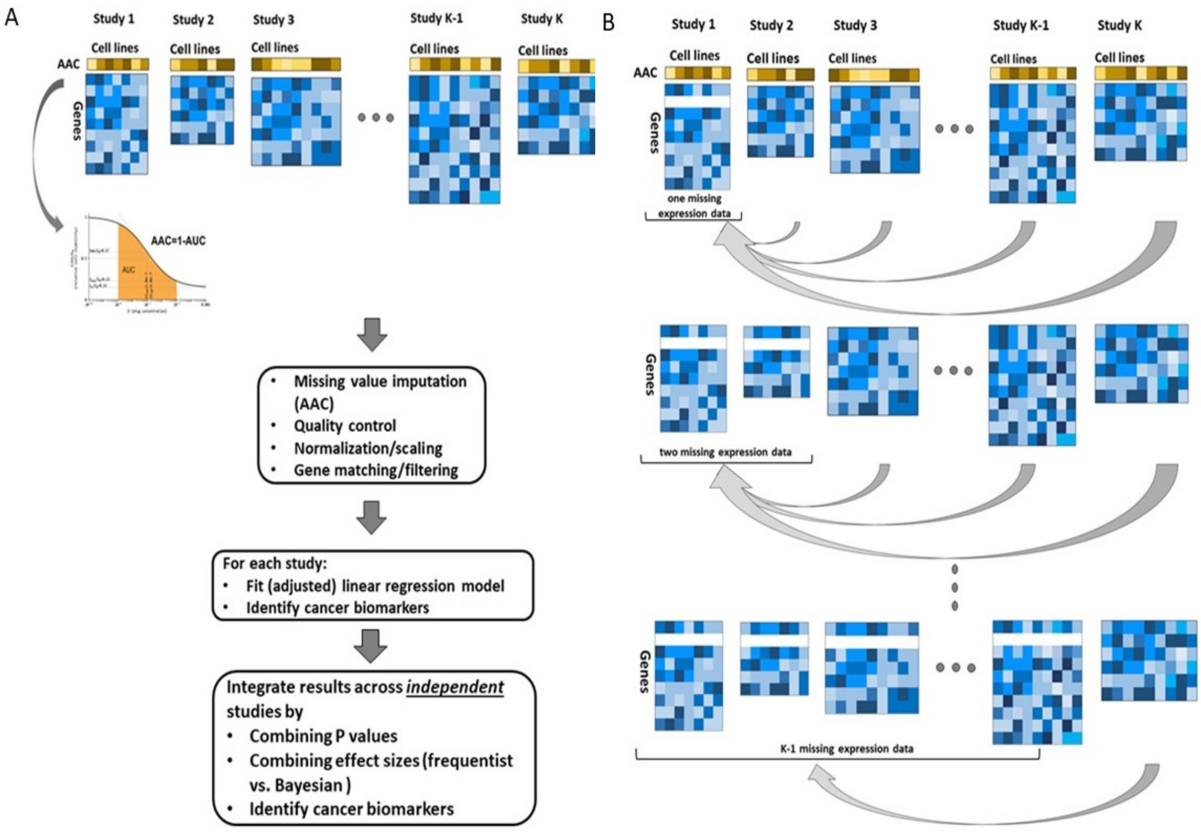

Figure S2. Flow diagram of meta-analysis. Integration analyses using (**A**) independent studies. (**B**) Duplicate partial or full missing expression data.

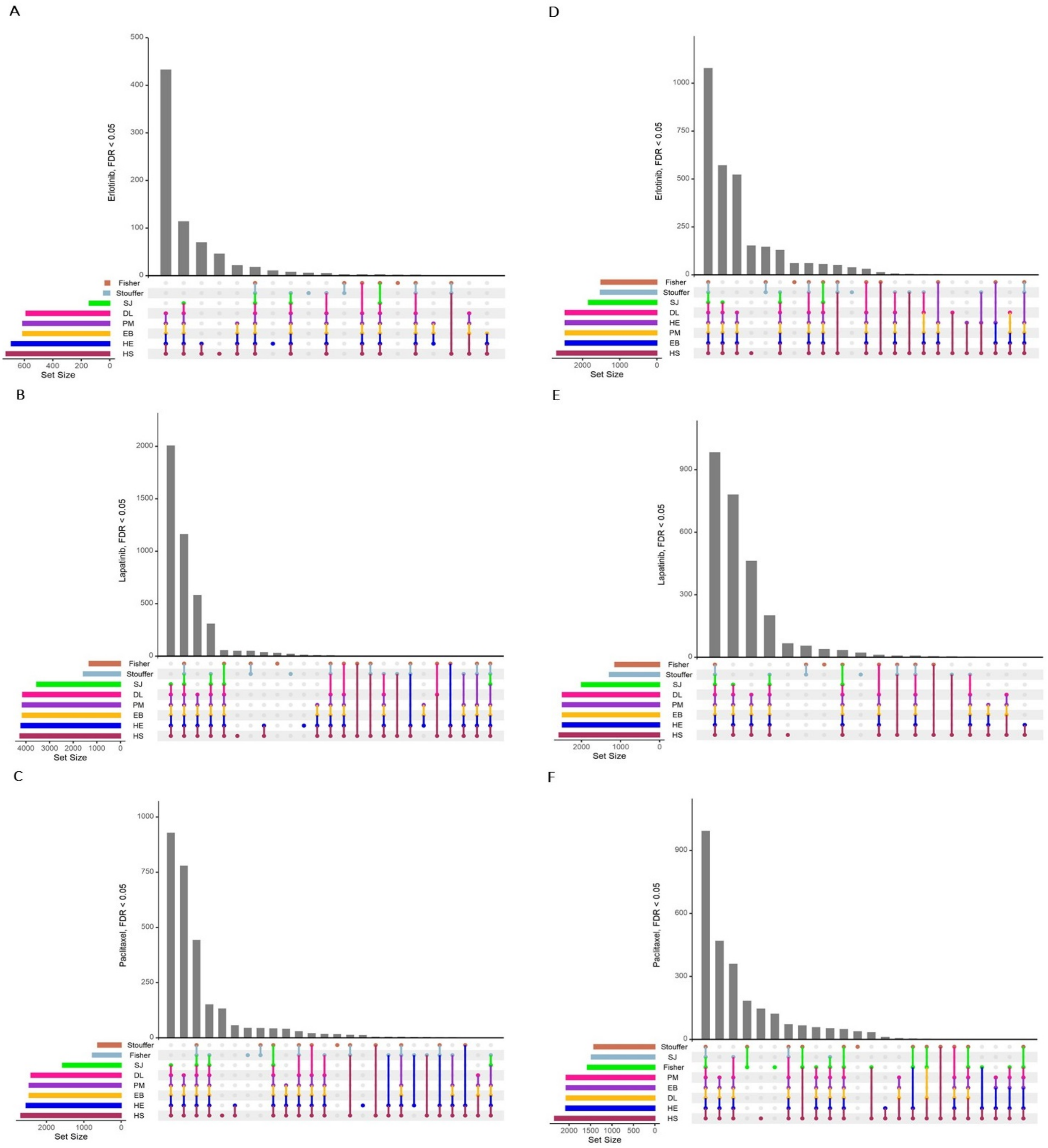

Figure S3. Comparison of different meta-analysis approaches. Upset diagrams represent the number of statistically significant genes associated with drug response (FDR < 0.05) using various meta-analysis methods for (**A-C**) breast and (**D-F**) pan-cancer data.

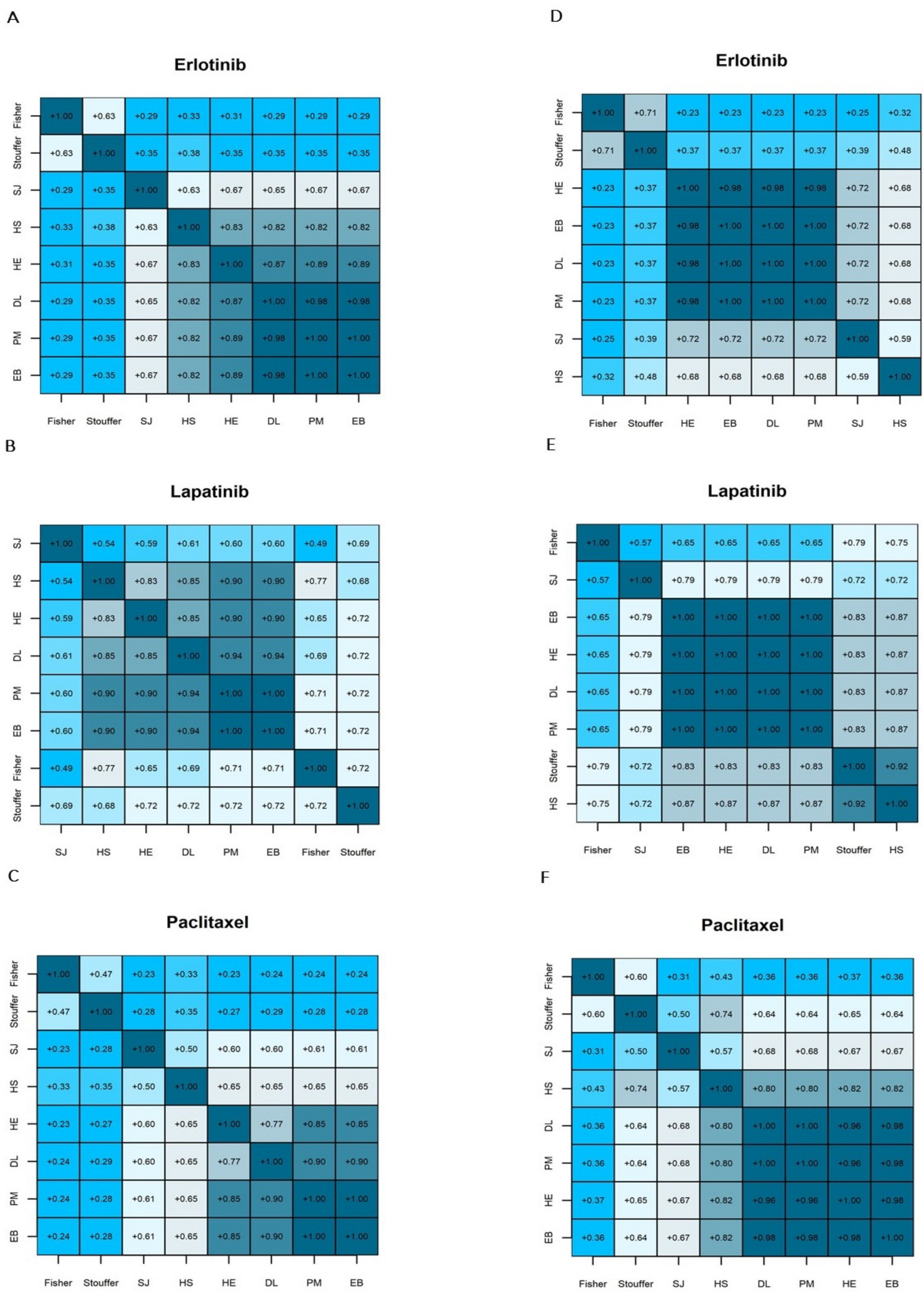

Figure S4. Stability metric to compare different meta-analysis approaches. Stability of identified 100 top-ranked genes associated with drugs by applying Jaccard similarity index using (**A-C**) breast and (**D-F**) pan-cancer data.

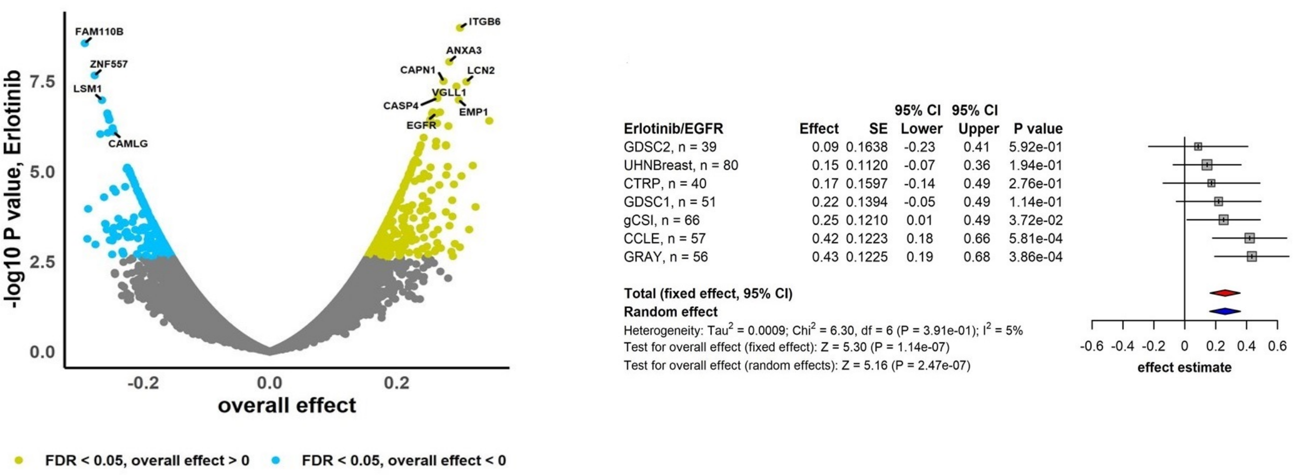

Figure S5. Breast cancer independent meta-analyses not limited to common cell lines across studies. Volcano plots show genes associated with drug response using RE meta-analysis model and forest plots illustrate overall effect estimate using FE (red diamond) and RE (blue diamond) meta-analysis models. DL approach was applied to estimate heterogeneity across studies. pan-cancer data.

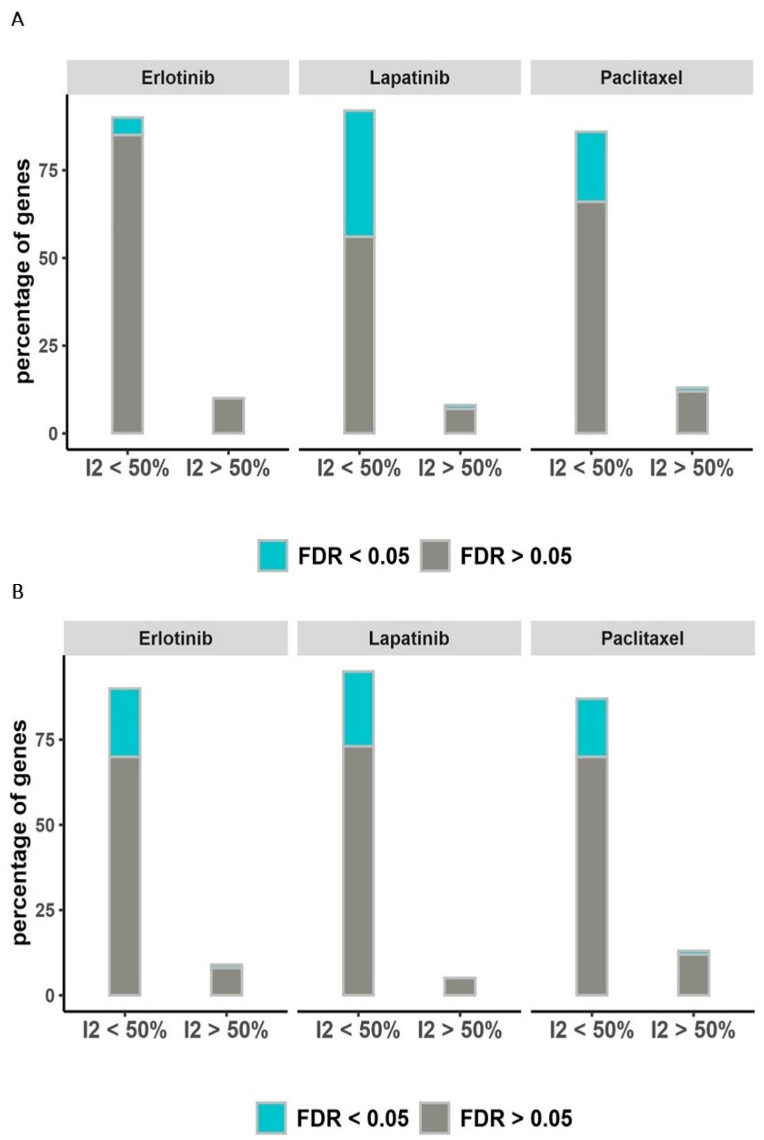

Figure S6. Gene-drug association meta-analyses and estimated heterogeneity. Bar plots present the percentage of gene-drug association meta-analyses using RE meta-analysis across estimated heterogeneity using (**A**) breast and (**B**) pan-cancer data.

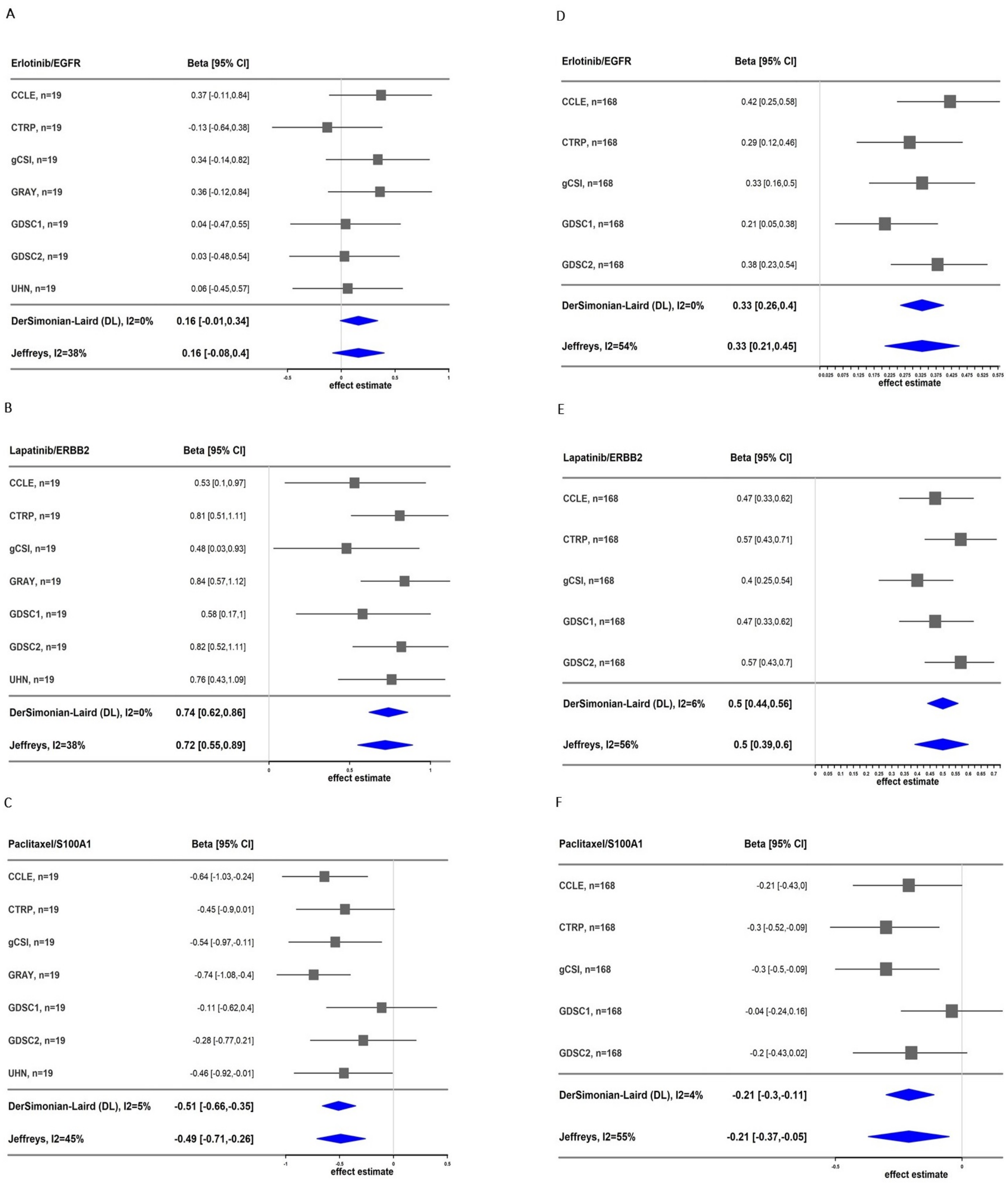

Figure S7. Bayesian versus classical independent meta-analyses. Forest plots illustrate overall effect estimate and 95% confidence and credible intervals along with heterogeneity estimate I^2 using DL and Bayesian Jeffreys procedures using (**A-C**) breast and (**D-F**) pan-cancer data.

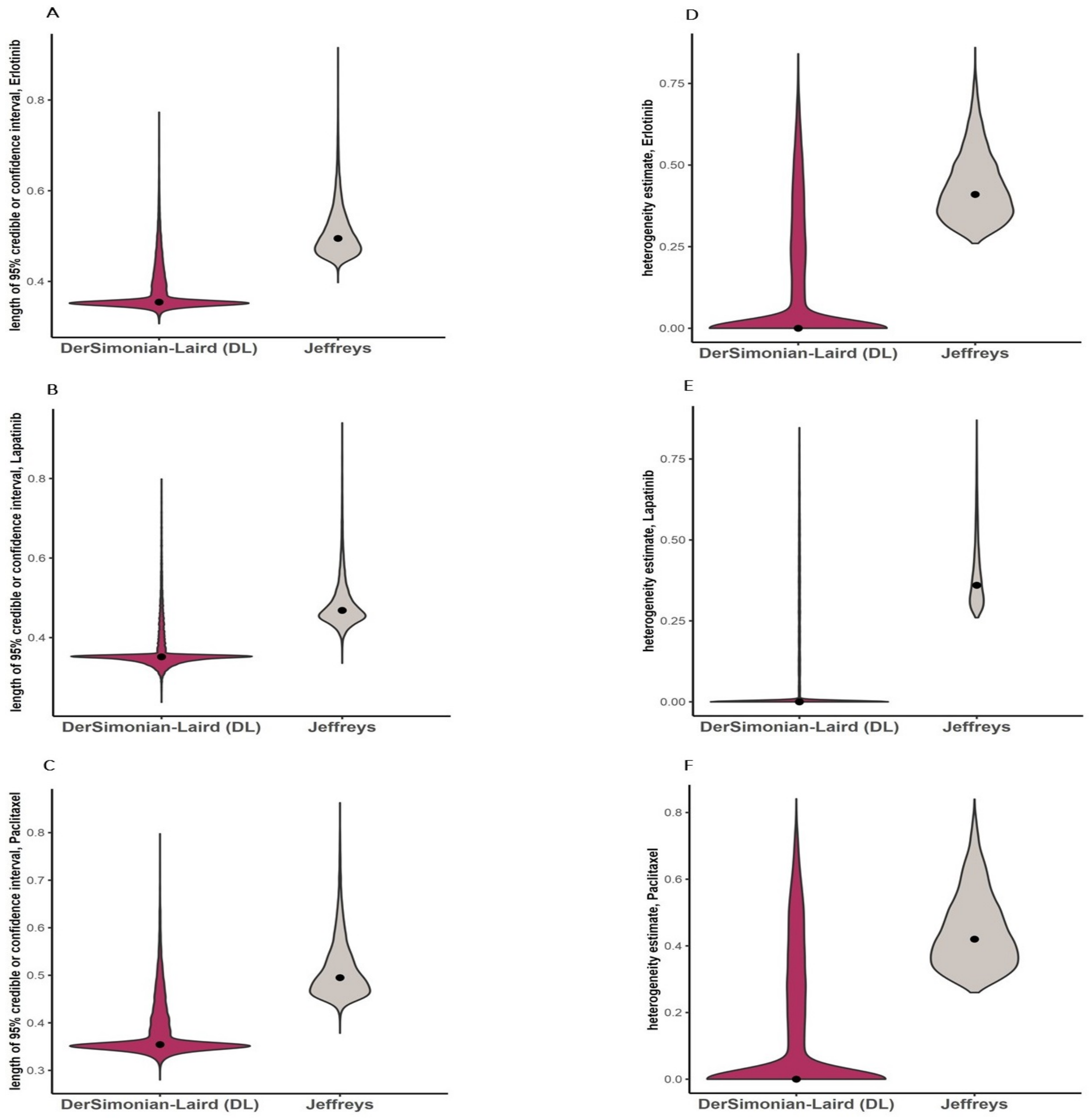

Figure S8. Bayesian versus classical independent to assess gene-drug association using breast cancer data. Violin plots illustrate (**A-C**) length of 95% confidence and credible intervals and (**D-F**) heterogeneity estimate I^2 using DL and Bayesian Jeffreys procedures. Black dot represents the median.

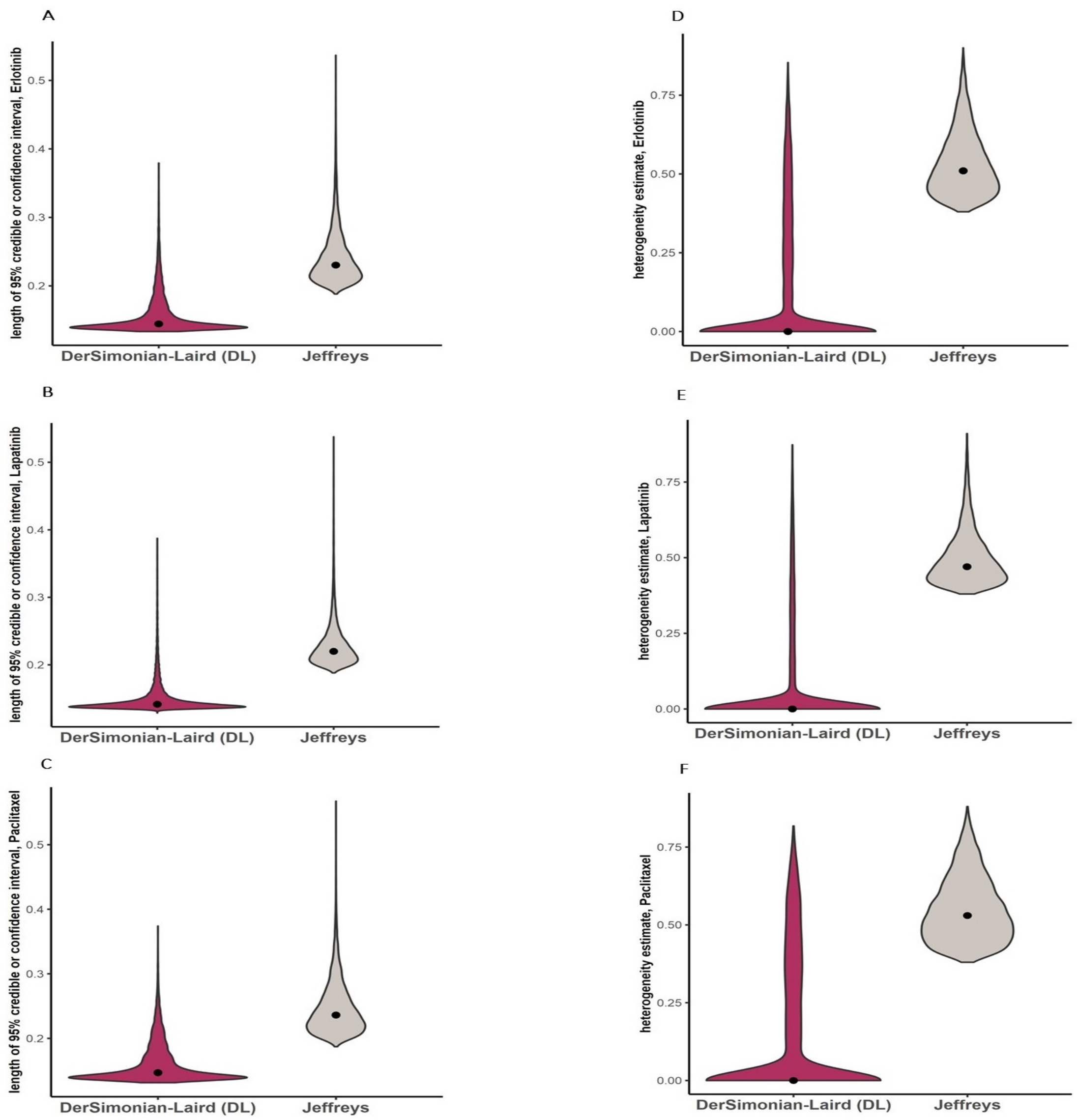

Figure S9. Bayesian versus classical independent to assess gene-drug association using pan-cancer data. Violin plots illustrate (**A-C**) length of 95% confidence and credible intervals and (**D-F**) heterogeneity estimate I^2 using DL and Bayesian Jeffreys procedures. Black dot represents the median.

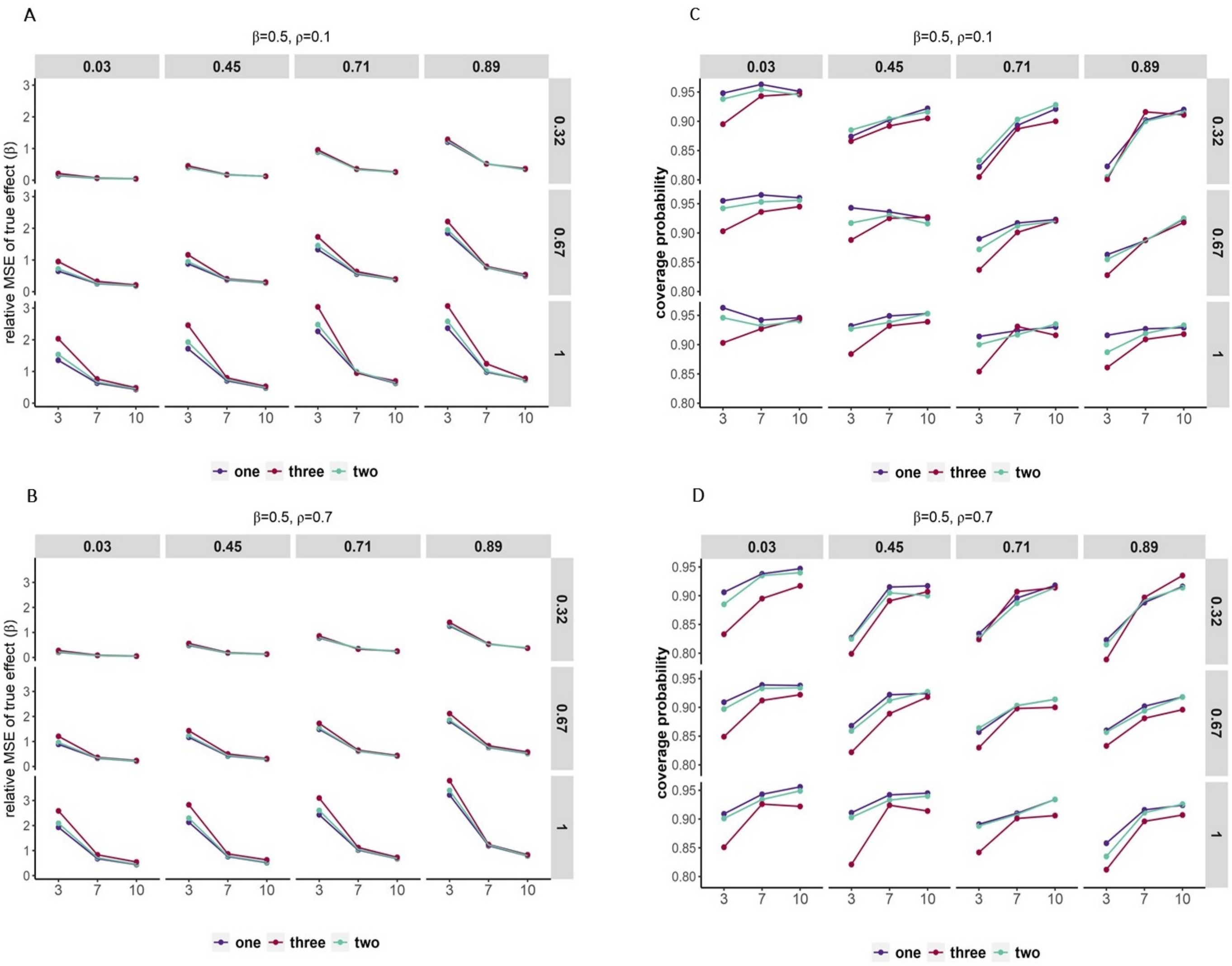

Figure S10. Mean squared error and coverage probability of overall effect estimates. Scenarios containing various within-study variances (row) and heterogeneity across studies (column). The x-axis represents the number of studies and the y-axis shows the relative MSE (**A-B**) and coverage probability of 95% confidence intervals (**C-D**). Set overall effect β = 0.5. Different colors represent a number of duplication or non-independent effects across studies.

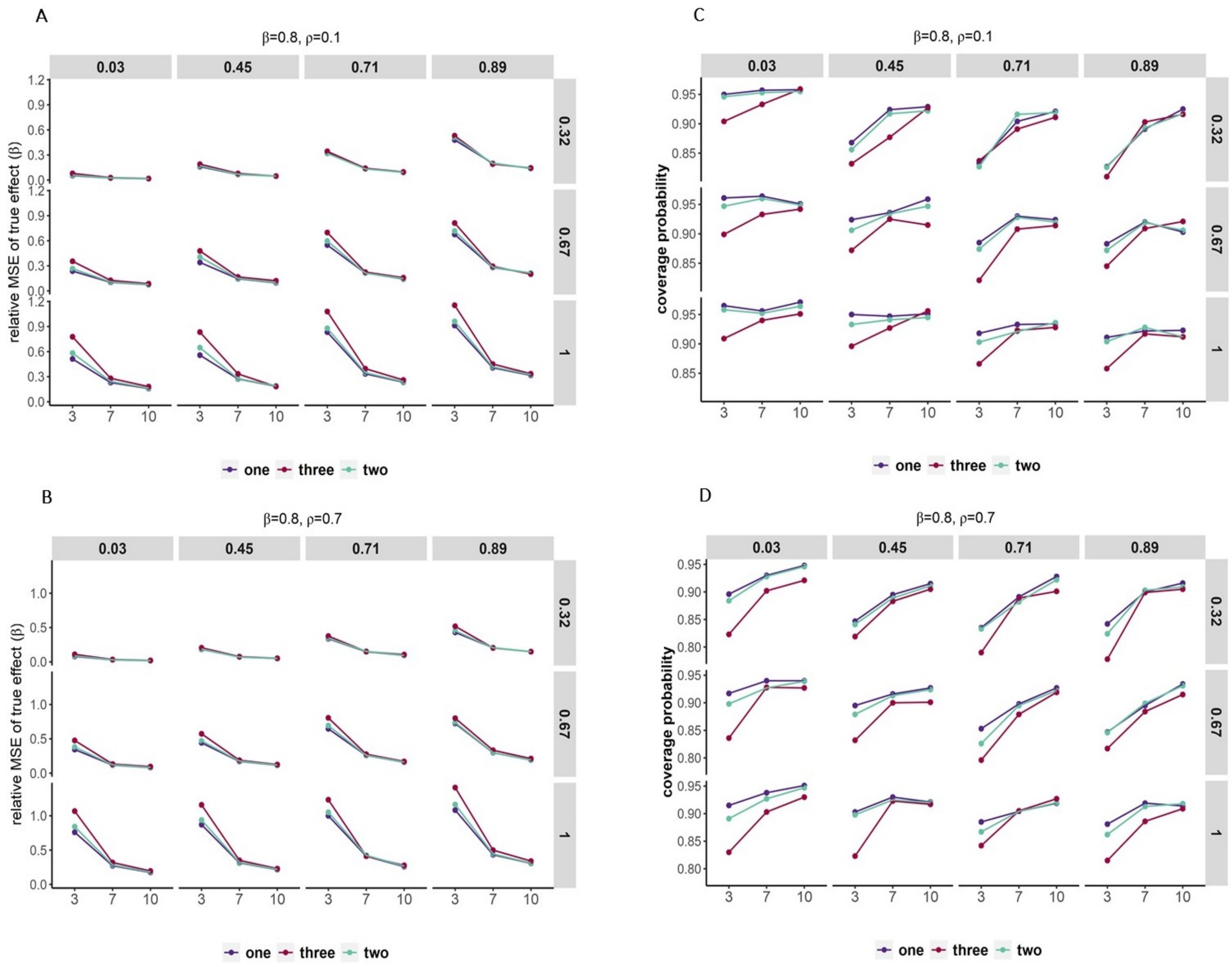

Figure S11. Mean squared error and coverage probability of overall effect estimates. Scenarios containing various within-study variances (row) and heterogeneity across studies (column). The x-axis represents the number of studies and the y-axis shows the relative MSE (**A-B**) and coverage probability of 95% confidence intervals (**C-D**). Set overall effect β = 0.8. Different colors represent a number of duplication or non-independent effects across studies.

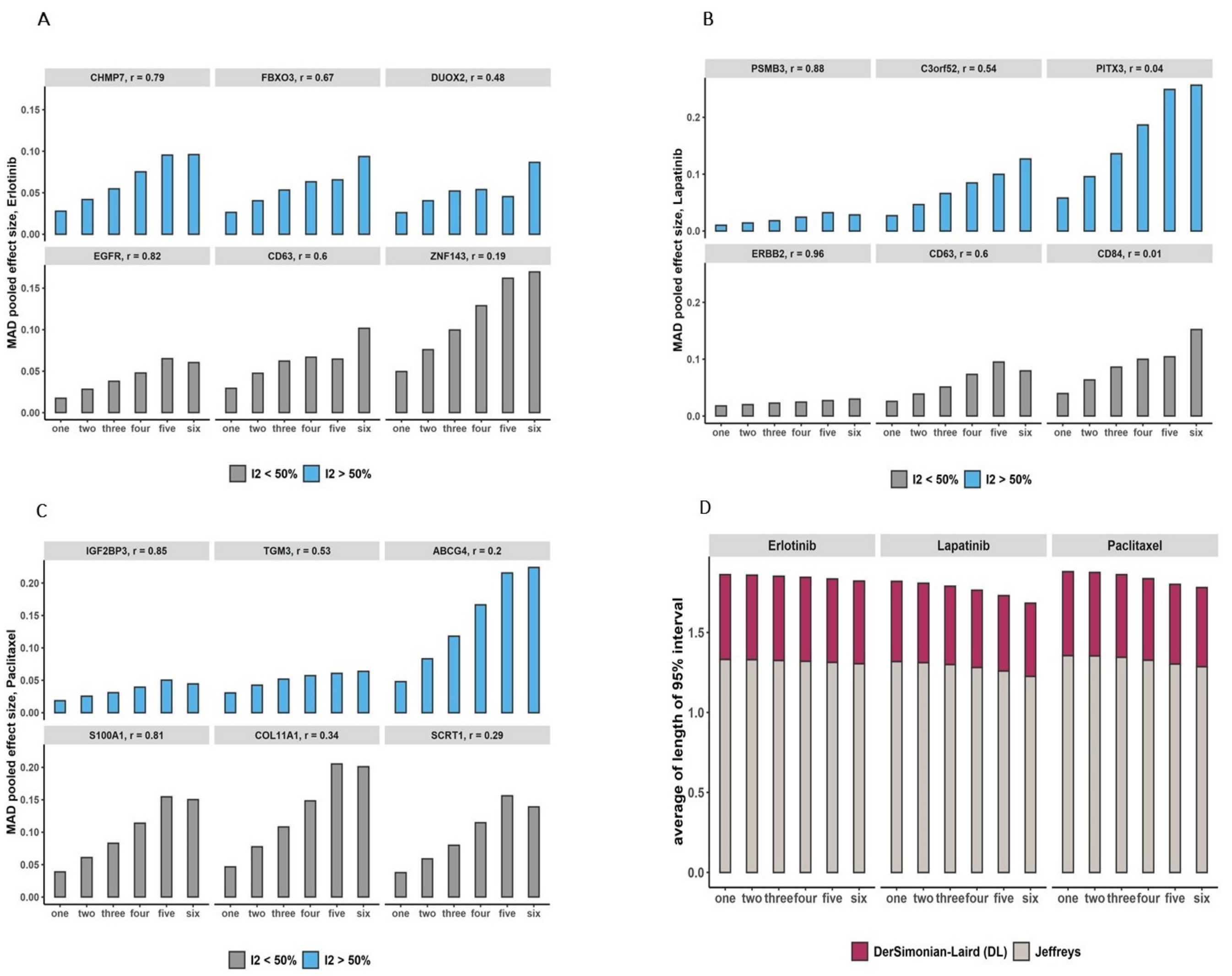

Figure S12. Breast cancer non-independent Jeffreys Bayesian meta-analyses. (**A-C**) Bar plots present increases in the number of duplications and its impact on the estimated overall effect using MAD metric across drugs and selected genes with substantial (blue) and non-substantial (gray) heterogeneity estimation. Note that x-axis presents the number of duplicate study effects. (**D**) Bar plots illustrate an average of 95% confidence or credible intervals for specific genes over the number of duplications using DL and Bayesian Jeffreys procedures.

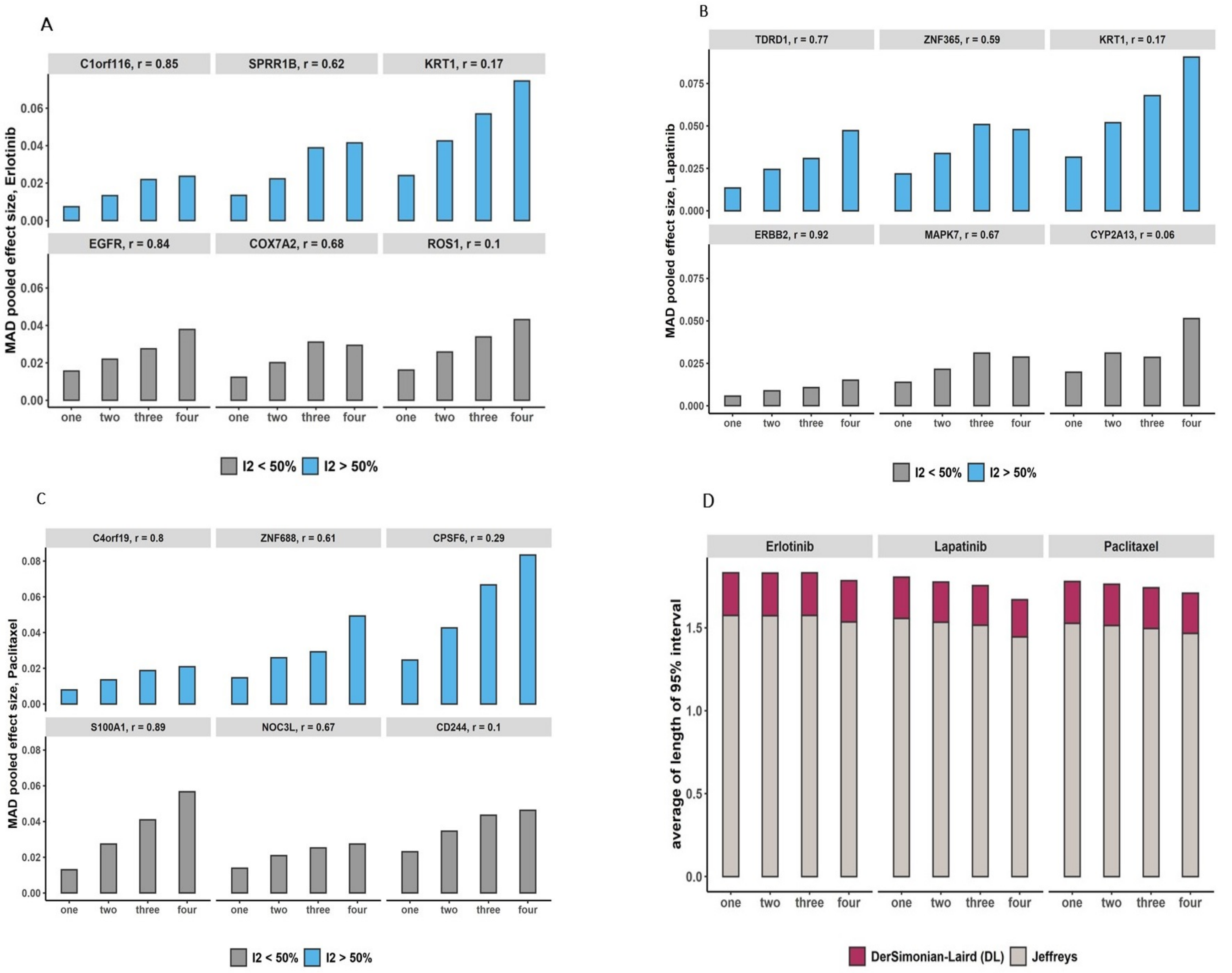

Figure S13. Pan-cancer non-independent Jeffreys Bayesian meta-analyses. (**A-C**) Bar plots present increases in the number of duplications and its impact on the estimated overall effect using MAD metric across drugs and selected genes with substantial (blue) and non-substantial (gray) heterogeneity estimation. Note that x-axis presents the number of duplicate study effects. (**D**) Bar plots illustrate an average of 95% confidence or credible intervals for specific genes over the number of duplications using DL and Bayesian Jeffreys procedures.
